# Dimeric PKM2 in chondrocytes impairs mitochondrial homeostasis in osteoarthritis

**DOI:** 10.1038/s41419-026-08621-4

**Published:** 2026-03-25

**Authors:** Bo Liu, Yun Liang, Chenzhong Wang, Ziyu Weng, Yi Yang, Yi Shi, Chi Zhang

**Affiliations:** 1 Department of Orthopedic Surgery, Zhongshan Hospital, Fudan University, Shanghai, China; 2 Department of Kidney Transplantation, Zhongshan Hospital, Fudan University, Shanghai, China; 3 Shanghai Key Laboratory of Organ Transplantation, Zhongshan Hospital, Fudan University, Shanghai, China

**Keywords:** Diseases, Molecular biology, Mechanisms of disease

## Abstract

Cartilage degradation is considered a hallmark of end-stage osteoarthritis (OA), characterized by significant alterations in the extracellular matrix (ECM). This study examines the role of pyruvate kinase muscle type 2 (PKM2) dimerization in cartilage degradation and ECM homeostasis in OA. Bioinformatic analyses identified an upregulation of PKM in OA cartilage, particularly within fibrocartilage subpopulations. Elevated expression and dimerization of PKM2 were observed in both human and murine OA cartilage. Chondrocyte-specific PKM2 deficiency, along with treatment using TEPP-46, a PKM2 tetramer stabilizer, reduced OA progression and promoted cartilage matrix production in a murine OA model with destabilization of the medial meniscus (DMM). Mechanistically, PKM2 deficiency or tetramer stabilization promoted mitochondrial fusion and preserved mitochondrial function via disruption of PKM2–ERK interaction, resulting in ERK-dependent upregulation of mitofusin 1 (MFN1), but not mitofusin 2 (MFN2). Notably, AAV-mediated MFN1 knockdown abrogated the chondroprotective effects of PKM2 deficiency. These findings indicate that targeting PKM2 dimerization may represent a promising therapeutic strategy for mitigating OA.

**Figure:**
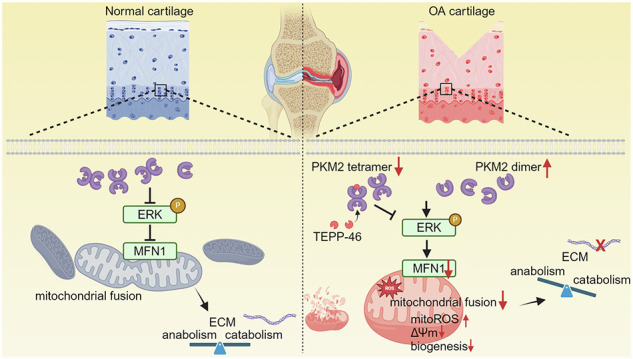
Increased PKM2 dimerization in osteoarthritic cartilage plays a pivotal role in extracellular matrix (ECM) degradation during osteoarthritis progression. Stabilization of PKM2 tetramers by TEPP-46 or genetic deletion of PKM2 disrupts PKM2–ERK interaction, promotes upregulation of the mitochondrial fusion protein MFN1, preserves mitochondrial function, and restores ECM homeostasis.

Increased PKM2 dimerization in osteoarthritic cartilage plays a pivotal role in extracellular matrix (ECM) degradation during osteoarthritis progression. Stabilization of PKM2 tetramers by TEPP-46 or genetic deletion of PKM2 disrupts PKM2–ERK interaction, promotes upregulation of the mitochondrial fusion protein MFN1, preserves mitochondrial function, and restores ECM homeostasis.

## Introduction

Osteoarthritis (OA) is a prevalent chronic joint disorder marked by cartilage degeneration, subchondral bone sclerosis, osteophyte formation, synovial inflammation, and alterations in ligaments and muscle structures [1, 2]. The articular cartilage consists of chondrocytes and the extracellular matrix (ECM) produced by these cells, mainly collagen type II alpha 1 chain (COL2A1) and aggrecan (ACAN) [3]. Together, COL2A1 and ACAN confer mechanical strength, enabling cartilage to resist compressive loading. Thus, cartilage degradation is a hallmark of OA, manifested by decreased ECM production resulting from increased expression of matrix metalloproteinases (MMPs) and a disintegrin and metalloproteinase with thrombospondin motifs (ADAMTSs) [4].

Glycolysis is the primary energy-producing pathway in chondrocytes, driven by the avascular and hypoxic environment of cartilage [5]. Pyruvate kinase muscle type (PKM) is the key rate-limiting enzyme in glycolysis, facilitating the conversion of phosphoenolpyruvate to pyruvate, and thereby generating adenosine triphosphate (ATP) [6–8]. The two isoforms of PKM, PKM1 and PKM2, generated through alternative splicing of the PKM gene, are widely expressed across different tissues [6, 9]. PKM1 is predominantly found in adult tissues, including muscle and brain, whereas PKM2 is mainly expressed in embryonic and tumor cells [10], suggesting that PKM2 plays a critical role under pathological conditions. Previous studies have established a multifaceted role for PKM2 in the pathogenesis of OA. PKM2 is markedly upregulated in OA chondrocytes and functions as a key regulator of glycolytic metabolism, thereby influencing cellular energy production and extracellular matrix synthesis [11]. Beyond its metabolic role, PKM2 has been implicated in the induction of endoplasmic reticulum stress, apoptosis, and inflammatory responses in chondrocytes, largely through signaling pathways such as Rspo2/Wnt/β-catenin [12]. In addition, PKM2 activity can be modulated by upstream regulatory networks, including the lncRNA PVT1/miR-552-3p competing endogenous RNA axis, which drives glycolytic flux and inflammation [13]. PKM2 has also been linked to mitochondrial quality control via the SIRT3–PINK1 pathway, thereby regulating mitophagy and metabolic reprogramming [14]. Notably, our previous work revealed that PKM2 regulates chondrocyte senescence through p16^INK4a^ in an inflammation-independent manner, underscoring the context-dependent functions of PKM2 in OA pathobiology [15]. Structurally, PKM2 exists in two conformations, dimeric and tetrameric states. The dimeric form of PKM2 can translocate to mitochondria or the nucleus, indicating its regulatory role in mitochondrial function and gene transcription [16, 17]. The tetrameric PKM2 exhibits enhanced metabolic activity in comparison to the dimeric form [18].

Metabolic changes in cells occur rapidly in response to physiological and pathological stimuli. More importantly, metabolic switches often precede functional changes and disease progression [19]. Mitochondria play a vital role in regulating cellular function and survival [20–22]. The equilibrium between mitochondrial fission and fusion is a crucial mechanism underlying the progression of OA [23, 24]. Mitochondrial fusion is controlled by mitofusins 1 and 2 (MFN1 and MFN2) and optic atrophy 1 (OPA1), whereas mitochondrial fission is regulated by dynamin-related protein 1 (DRP1) [23]. It has been reported that increased MFN2 levels contribute to inflammation and senescence in OA [25, 26], whereas overexpression of MFN2 has been shown to decelerate the progression of OA via the NOTCH2 pathway [27].

Although previous studies have implicated PKM2 in OA pathogenesis, such as its involvement in glycolysis, endoplasmic reticulum stress, and senescence, the specific role of PKM2 dimerization in mitochondrial dynamics remains unexplored. Herein, we identify dimeric PKM2 as a critical factor in the progression of OA. Reduced PKM2 dimerization, achieved through genetic modification and pharmacological approaches, regulates MFN1-mediated mitochondrial fusion via the extracellular signal-regulated kinase (ERK) pathway. These findings imply that targeting PKM2 dimerization represents a promising strategy for OA treatment.

## Results

### PKM2 expression is increased in osteoarthritic cartilage

**Fig. 1:**
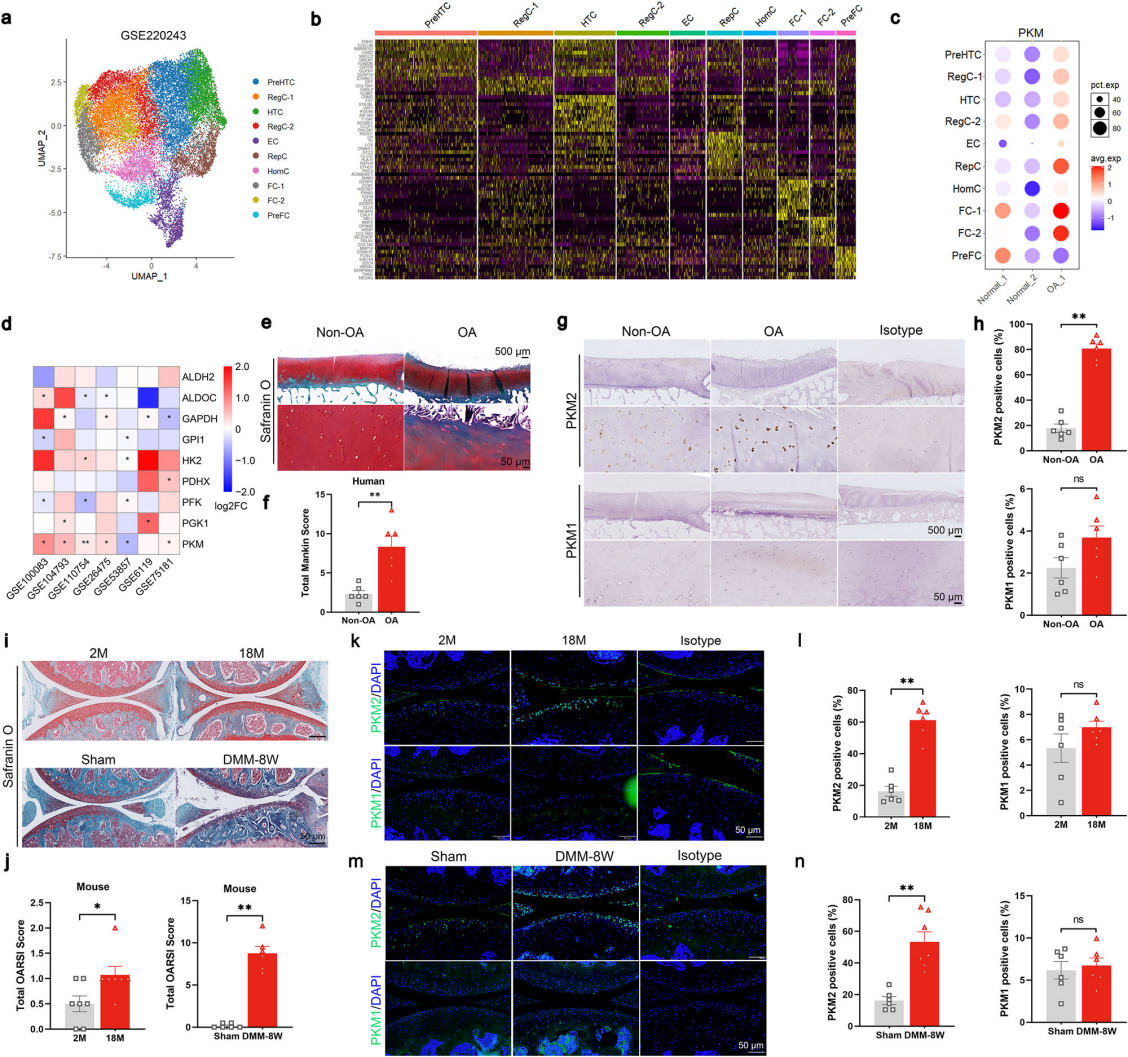
PKM2 plays a critical role in the development of osteoarthritis. **a** Uniform manifold approximation and projection (UMAP) visualization of public single-cell transcriptome data (GSE220243) from human OA cartilage. **b** Heatmap depicting the scaled expression of differentially expressed genes across subclusters. **c** Dot plots illustrating *PKM* expression levels in distinct cell subclusters. **d** Heatmap depicting the transcriptional profiles of glycolytic enzymes in seven publicly available OA-related datasets. FC, fold change. **e** Representative images of SO&FG staining and **f** quantification of Mankin scores in cartilage samples collected from non-OA and OA patients. **g** Representative images of immunohistochemical (IHC) staining and **h** quantification of PKM1 and PKM2 expression levels in human non-OA and OA cartilage. **i** Representative images of SO&FG staining and **j** quantification of Osteoarthritis Research Society International (OARSI) scores in cartilage from 2-month-old young mice and 18-month-old aged mice (upper panel), and from mice eight weeks after DMM or Sham surgery (lower panel). **k** Representative images of immunofluorescence (IF) staining and **l** quantification of PKM1 and PKM2 expression levels in cartilage from young and aged mice. **m** Representative IF images and **n** quantification of PKM1 and PKM2 expression levels in cartilage from mice eight weeks post-DMM or Sham surgery. Data were presented as means ± s.e.m., *n* = 6, unpaired Student’s *t*-test. *P < 0.05, **P < 0.01, ***P < 0.001. ns not significant.

To investigate the involvement of glycolytic enzymes in OA pathogenesis, we analyzed a public single-cell RNA-seq (scRNA-seq) dataset (GSE220243) generated from cartilage tissue of one OA patient undergoing knee arthroplasty and two non-OA donors [28]. Ten chondrocyte subpopulations were identified, including prehypertrophic chondrocytes (PreHTC), regulatory chondrocytes (RegC, comprising RegC-1 and RegC-2), hypertrophic chondrocytes (HTC), effector chondrocytes (EC), reparative chondrocytes (RepC), homeostatic chondrocytes (HomC), fibrocartilage chondrocytes (FC, comprising FC-1 and FC-2), and pre-fibrocartilage chondrocytes (PreFC) (Fig. 1a, b). Among glycolytic enzymes, PKM emerged as the sole molecule consistently upregulated in OA cartilage (Fig. 1c and Supplementary Fig. 1a). Among the subpopulations, PKM expression was highest in the FC-2 subcluster, which regulates angiogenesis, ossification and cartilage fibrosis, and lowest in the RegC-2 subgroup, which is involved in chondrogenic differentiation [29] (Fig. 1c and Supplementary Fig. 1b). Notably, the FC-2 subgroup displayed the greatest expression of type I collagen (COL1A1 and COL1A2), major structural components of fibrocartilage, while the RegC-2 subgroup exhibited the highest expression of COL2A1, the principal collagen of hyaline cartilage (Supplementary Fig. 1b). When comparing FC-2 and RegC-2 cells, the RegC-2 subgroup demonstrated enhanced expression of extracellular matrix (ECM)-related genes including COL2A1, ACAN, proteoglycan 4 (PRG4), collagen type XI alpha 2 chain (COL11A2), cartilage oligomeric matrix protein (COMP), and SRY-box transcription factor 9 (SOX9), accompanied by reduced expression of ECM-degrading enzymes such as MMP3 and ADAMTS5 (Supplementary Fig. 1c). Because fibrocartilaginous transformation reflects progressive OA pathology, the enrichment of PKM in FC-2 suggested that PKM may be linked to ECM metabolism. Increased PKM expression in OA cartilage was further validated using seven publicly available OA-related transcriptomic datasets (Fig. 1d). Together, both single-cell and bulk RNA-seq data consistently demonstrated transcriptional upregulation of PKM in OA cartilage, suggesting a possible association between PKM levels and ECM homeostasis.

PKM1 and PKM2 are splice isoforms of the PKM gene, exhibiting distinct expression patterns across tissues. To further define PKM expression, we collected cartilage samples from six OA patients undergoing total knee arthroplasty and six non-OA controls. Compared with normal cartilage, human OA cartilage showed markedly reduced expression of COL2A1 and ACAN, along with increased MMP13 expression (Supplementary Fig. 2a, b). Safranin O–fast green (SO&FG) staining demonstrated decreased proteoglycan content with higher total Mankin scores in OA cartilage (Fig. 1e, f). Notably, PKM2, but not PKM1, was elevated in OA cartilage (Fig. 1g, h and Supplementary Fig. 2c). We next examined PKM2 expression in cartilage from surgically induced (DMM) and aging-associated OA mouse models. In both models, SO&FG staining and Osteoarthritis Research Society International (OARSI) scoring confirmed the cartilage degeneration (Fig. 1i, j). Increased expression of p16^INK4a^ and p21, both established markers of cellular senescence, was also observed (Supplementary Fig. 2d–g). There was decreased expression of COL2A1 and ACAN in cartilage from mice eight weeks after the DMM surgery (Supplementary Fig. 2d, e). Consistent with human OA, PKM2 expression was significantly elevated in articular cartilage from both mouse models, whereas PKM1 expression was barely detected (Fig. 1k–n). Under high magnification, nuclear PKM2 signals were observed in chondrocytes in both models (Supplementary Fig. 2h, i). Moreover, PKM2 expression was elevated in osteoarthritic meniscus, as shown by immunofluorescence (IF) staining and supported by analysis of a bulk RNA-seq dataset (GSE241794) (Supplementary Fig. 3). Collectively, these findings indicate that PKM2, but not PKM1, is upregulated in OA cartilage and may contribute to OA progression.

### Pkm2 knockdown restores homeostasis of matrix metabolism in osteoarthritic cartilage

**Fig. 2:**
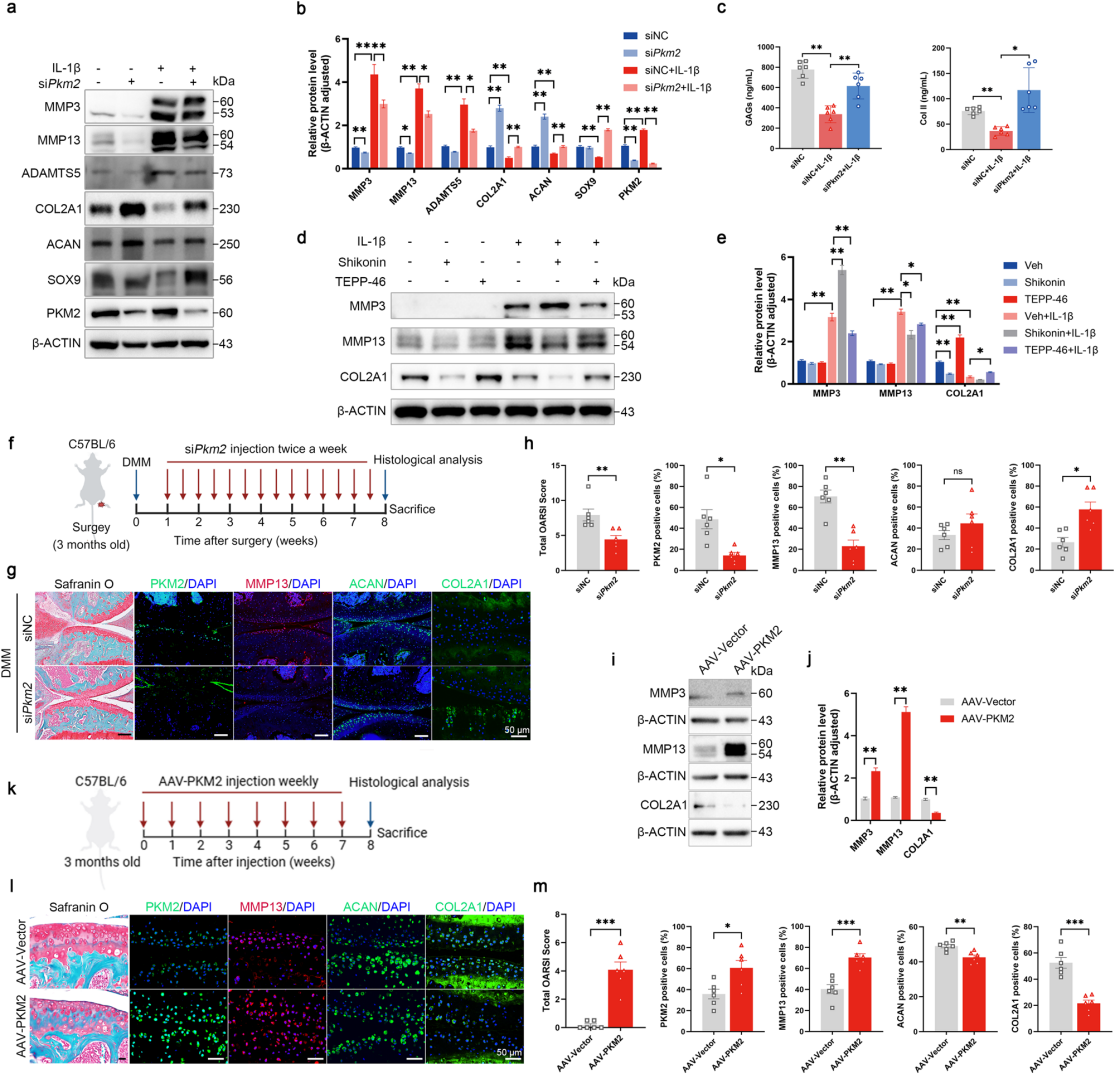
PKM2 regulates extracellular matrix (ECM) metabolism in chondrocytes. **a** Representative immunoblots and **b** densitometric quantification of MMP3, MMP13, ADAMTS5, COL2A1, ACAN, SOX9, and PKM2 in chondrocytes transfected with siNC or siPkm2 and stimulated with IL-1β (1 ng mL⁻¹). Data were presented as means ± s.e.m., *n* = 6, one-way ANOVA with Tukey’s multiple comparisons. **c** Quantification of type II collagen (Col II) and glycosaminoglycans (GAGs) in the culture supernatant of chondrocytes transfected with siNC or siPkm2 and stimulated with IL-1β (10 ng mL⁻¹). Data were presented as means ± s.e.m., *n* = 6, one-way ANOVA with Tukey’s multiple comparisons. **d** Representative immunoblots and **e** densitometric quantification of MMP3, MMP13, and COL2A1 in IL-1β-stimulated chondrocytes incubated with vehicle, Shikonin (1 μM) or TEPP-46 (50 μM). Data were presented as means ± s.e.m., *n* = 6, one-way ANOVA with Tukey’s multiple comparisons. **f** Schematic of intra-articular siNC or siPkm2 administration in DMM mice. Three-month-old WT mice underwent DMM surgery, and intra-articular injections of siNC or siPkm2 were initiated one week later and administered twice weekly for eight weeks. Mice were euthanized eight weeks post-surgery, and cartilage was collected for histological analysis. **g** Representative images of SO&FG staining and IF staining, and **h** quantification of PKM2, MMP13, ACAN, and COL2A1 expression in cartilages from DMM mice administered with siNC or siPkm2. Data were presented as means ± s.e.m., *n* = 6, unpaired Student’s *t*-test. **i** Representative immunoblots and **j** densitometric quantification of MMP3, MMP13, and COL2A1 in chondrocytes transfected with AAV-Vector or AAV-PKM2. Data were presented as means ± s.e.m., *n* = 6, unpaired Student’s *t*-test. **k** Schematic diagram of intra-articular AAV-Vector or AAV-PKM2 administration. Three-month-old WT mice received intra-articular injections once weekly for 8 weeks; mice were then euthanized and cartilage harvested for histological analysis. **l** Representative SO&FG and IF staining images, and **m** quantification of PKM2, MMP13, ACAN, and COL2A1 expression in cartilages from mice administered with AAV-Vector or AAV-PKM2. Data were presented as means ± s.e.m., *n* = 6, unpaired Student’s *t*-test. *P < 0.05, **P < 0.01, ***P < 0.001. ns not significant.

To further delineate the functional role of PKM2 in OA, three small interfering RNAs (siRNAs) targeting Pkm2 were designed and transfected into primary mouse chondrocytes (Supplementary Fig. 4a). The knockdown efficiency was validated via quantitative reverse transcription polymerase chain reaction (qRT-PCR) and western blotting (Supplementary Fig. 4b–d). We next investigated the role of PKM2 reduction on ECM metabolism. Under basal conditions, Pkm2 knockdown significantly decreased the mRNA and protein expression of ECM-degrading enzymes, including MMP3, MMP13, and ADAMTS5, while increasing the expression of ECM structural components, such as COL2A1 and ACAN (Fig. 2a, b and Supplementary Fig. 4e). Treatment of cultured chondrocytes with interleukin-1 beta (IL-1β), a pro-inflammatory cytokine commonly used to mimic OA conditions, reduced the expression of ECM proteins COL2A1 and ACAN and increased the levels of catabolic markers MMP3, MMP13, and ADAMTS5 (Fig. 2a, b and Supplementary Fig. 4f). Importantly, Pkm2 knockdown reversed the IL-1β-induced downregulation of COL2A1 and ACAN and suppressed the upregulation of MMP3, MMP13, and ADAMTS5 (Fig. 2a, b and Supplementary Fig. 4f). Moreover, SOX9, a pivotal regulator of cartilage development and chondrocyte differentiation, was markedly elevated in Pkm2-knockdown cells following IL-1β exposure (Fig. 2a, b). Consistent with these findings, the protein levels of type II collagen and glycosaminoglycans were increased in the conditioned medium of IL-1β-treated cells after Pkm2 knockdown (Fig. 2c). Pharmacological stabilization of PKM2 in its tetrameric conformation using TEPP-46 [30] induced COL2A1 expression while downregulating MMP3 and MMP13 (Fig. 2d, e). Conversely, Shikonin, a PKM2 inhibitor [31], exhibited the opposite effects by markedly increasing MMP3 and MMP13 levels and suppressing COL2A1 expression (Fig. 2d, e). We next evaluated whether Pkm2 knockdown could mitigate OA progression in vivo. Following destabilization of the medial meniscus (DMM) surgery, siPkm2 was administered via intra-articular injection (Fig. 2f). SO&FG staining showed that siPkm2 treatment substantially attenuated cartilage degeneration and reduced OARSI scores (Fig. 2g, h). Additionally, Pkm2 knockdown suppressed MMP13 expression and enhanced COL2A1 expression in articular cartilage (Fig. 2g, h). In contrast, adeno-associated virus (AAV)–mediated PKM2 overexpression upregulated MMP3 and MMP13 while decreasing COL2A1 expression (Fig. 2i, j). Consistently, intra-articular AAV-PKM2 administration induced spontaneous cartilage degeneration, promoting ECM catabolism while suppressing ECM anabolism (Fig. 2k–m).

**Fig. 3:**
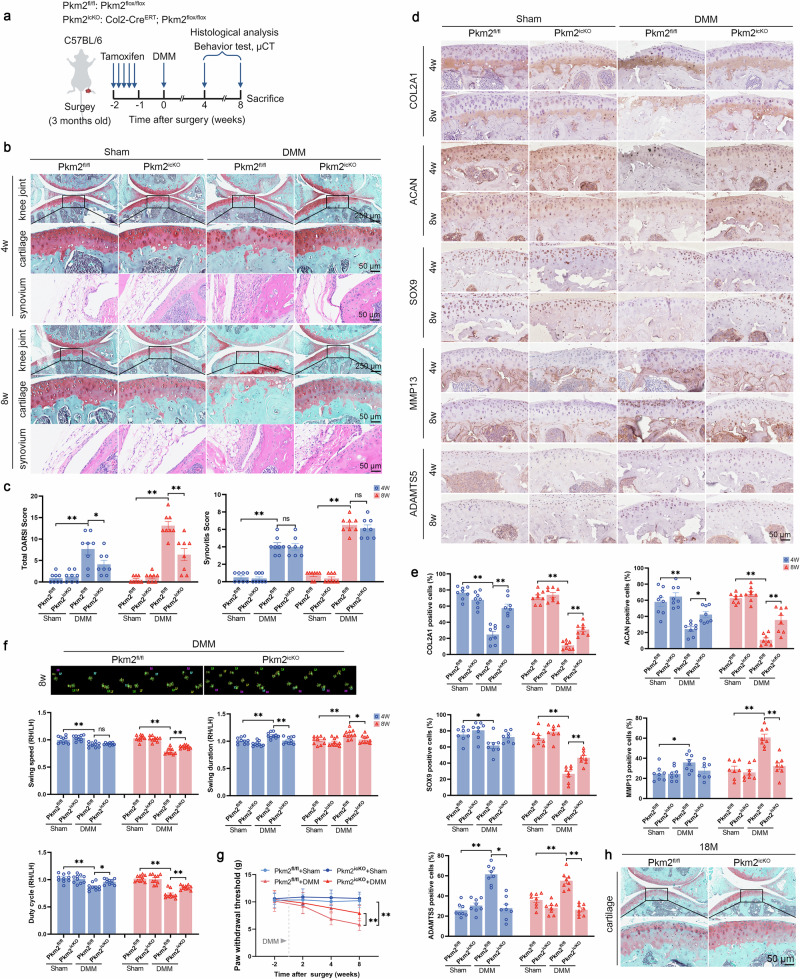
Chondrocyte-specific PKM2 deletion protects mice from DMM-induced and aging-related OA. **a** Schematic diagram of cartilage-specific Pkm2 deletion in DMM-induced OA mouse models. Three-month-old Pkm2^fl/fl^ and Pkm2^icKO^ mice received tamoxifen intraperitoneally for five consecutive days to induce cartilage-specific Pkm2 deletion, followed by DMM surgery. Mice were euthanized at four or eight weeks post-DMM for cartilage histology, hind-limb behavioral testing, and subchondral bone micro-CT. **b** Representative SO&FG staining of cartilages and HE staining of synovium in Pkm2^fl/fl^ and Pkm2^icKO^ mice at four weeks (upper panel) and eight weeks (lower panel) post-DMM. **c** Quantification of total OARSI scores (left) for cartilage and synovitis scores (right) in Pkm2^fl/fl^ and Pkm2^icKO^ mice at 4 weeks and 8 weeks post-DMM. **d** Representative IHC images and **e** quantification of COL2A1, ACAN, SOX9, MMP13, and ADAMTS5 expression in cartilage from Pkm2^fl/fl^ and Pkm2^icKO^ mice, four and eight weeks after DMM surgery. **f** CatWalk gait analysis showing representative paw-print traces and quantification of hind-limb movement parameters (swing speed, swing duration, and duty cycle), presented as right hind limb/left hind limb (RH/LH) ratios. **g** Paw withdrawal threshold measured by the von Frey test. **h** Representative SO&FG staining image of cartilages tissues from 18-month-old Pkm2^fl/fl^ and Pkm2^icKO^ mice. Data were presented as means ± s.e.m., *n* = 8, one-way ANOVA with Tukey’s multiple comparisons. *P < 0.05, **P < 0.01. ns not significant.

To further assess the protective role of PKM2 intervention during OA development, we utilized a CRISPR-Cas9 system to generate chondrocyte-specific PKM2 knockout mice (hereafter referred to as Pkm2^icKO^, with Pkm2^flox/flox^ as controls, hereafter referred to as Pkm2^fl/fl^) via the Col2-Cre^ERT^ transgene (Supplementary Fig. 5a–c). Western blotting and IF staining confirmed a marked reduction of PKM2, without alterations in PKM1 expression, in the articular cartilage of adult Pkm2^icKO^ compared with Pkm2^fl/fl^ controls (Supplementary Fig. 5d–g). Gross morphological examination indicated that PKM2 deletion during the developmental stage did not affect body length or limb development (Supplementary Fig. 5h, i). However, a moderate increase in PKM1 expression was detected in growth plates and articular cartilage of four-week-old Pkm2^icKO^ mice, whereas this compensatory response was absent in adult mice (Supplementary Fig. 5f, g, j). Four weeks after DMM surgery, Pkm2^fl/fl^ mice exhibited proteoglycan depletion, cartilage surface irregularities, and synovial hyperplasia, which progressed to extensive cartilage erosion, surface roughening, and pronounced synovial inflammation by 8 weeks (Fig. 3a–c). In contrast, Pkm2^icKO^ mice subjected to DMM surgery retained substantially better cartilage integrity, despite displaying a similar degree of synovial thickening (Fig. 3b, c). Because OA progression reflects a disruption in anabolic–catabolic balance and chondrocyte viability, we evaluated ECM-related markers and cellular apoptosis. At both four and eight weeks post-DMM, Pkm2^icKO^ mice exhibited increased protein levels of COL2A1, ACAN, and SOX9, along with decreased levels of MMP13 and ADAMTS5 in cartilage tissue, compared with Pkm2^fl/fl^ mice (Fig. 3d, e). Consistent with improved matrix homeostasis, Pkm2^icKO^ mice displayed elevated levels of proliferating cell nuclear antigen (PCNA) and reduced apoptotic signals (Supplementary Fig. 6a, b).

We previously reported that Pkm2 knockdown suppressed p16^INK4a^ expression and alleviated chondrocyte senescence in vitro [15]. Here, we further examined the effects of Pkm2 deficiency on senescence in vivo. The protein levels of p16^INK4a^ and p21 were increased in human and mouse OA cartilage (Supplementary Fig. 7a, b). However, these senescence markers were notably reduced in Pkm2^icKO^ mice compared with Pkm2^fl/fl^ mice eight weeks after DMM surgery (Supplementary Fig. 7a, b). Given the association between OA progression, pain, and aberrant subchondral bone remodeling, micro-CT scans were conducted to evaluate subchondral bone structure. Tibial subchondral bone sclerosis was alleviated in Pkm2^icKO^ mice, characterized by reduced trabecular bone volume fraction, while the thickness of the subchondral bone plate remained comparable (Supplementary Fig. 8a, b).

Since pain is a primary symptom of OA, we next employed CatWalk gait analysis and Von Frey testing to assess motor coordination and mechanical pain threshold, respectively. Compared with Pkm2^fl/fl^ mice, Pkm2^icKO^ mice exhibited significant improvements in swing speed, swing duration, and duty cycle in the CatWalk assay, with no differences in mean intensity or paw area (Fig. 3f and Supplementary Fig. 8c, d). Consistently, Von Frey testing revealed a higher paw withdrawal threshold in Pkm2^icKO^ mice, indicative of reduced mechanical hypersensitivity (Fig. 3g). We further examined cartilage integrity in 18-month-old Pkm2^fl/fl^ and Pkm2^icKO^ mice. Enhanced SO&FG staining intensity was observed in Pkm2^icKO^ mice (Fig. 3h). Collectively, these data suggest that Pkm2 deficiency in chondrocytes mitigates surgery-induced cartilage damage and alleviates OA-associated pain.

### PKM2 dimerization changes ECM metabolism in osteoarthritis

**Fig. 4:**
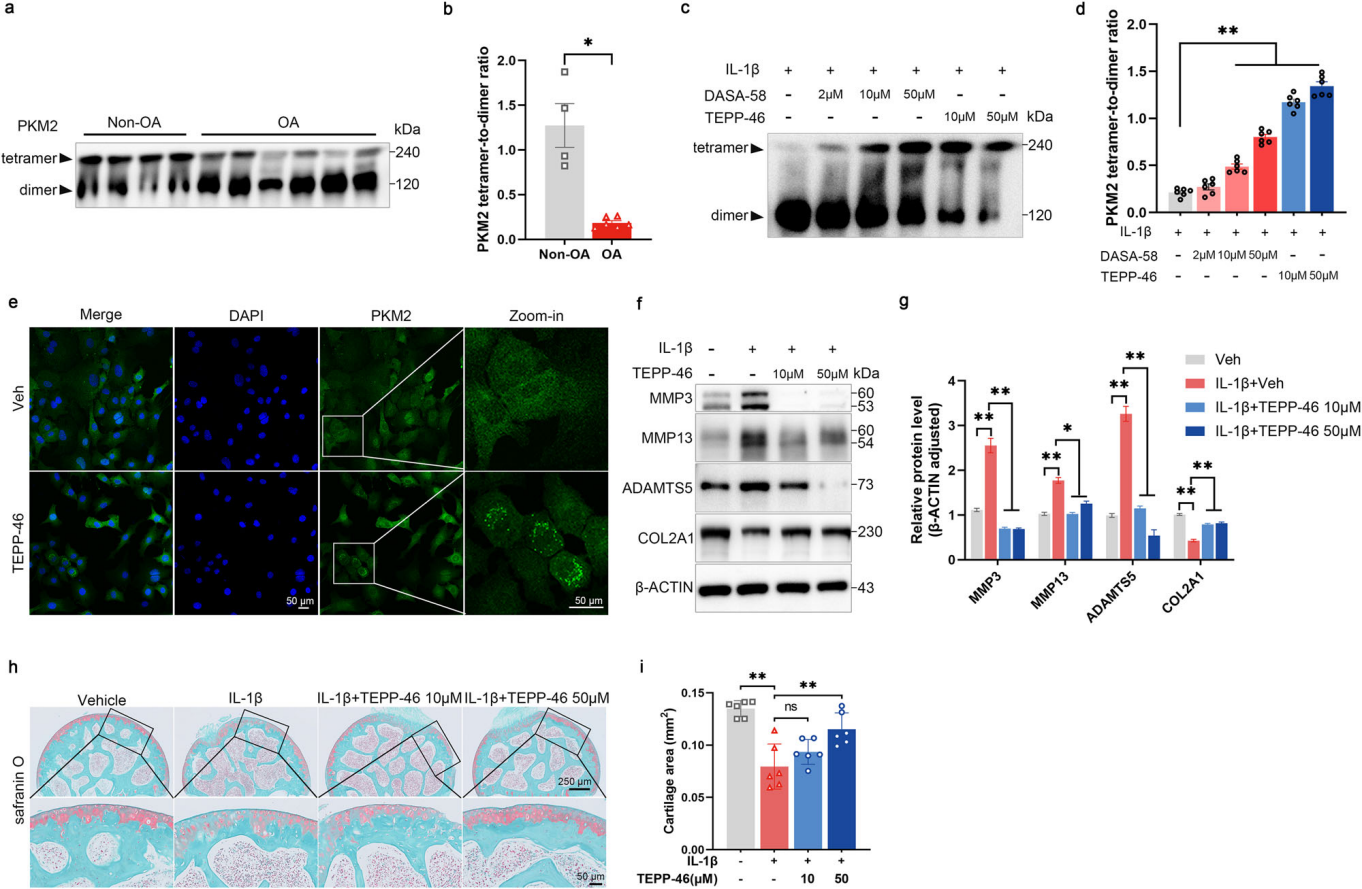
TEPP-46 inhibits PKM2 dimer formation and maintains ECM metabolism homeostasis in vitro. **a** Representative immunoblots and **b** densitometric quantification of tetrameric and dimeric PKM2 in cartilage tissues from non-OA (*n* = 4) and OA (*n* = 6) patients. Data were presented as means ± s.e.m., unpaired Student’s *t*-test. **c** Representative immunoblots and **d** densitometric quantification of tetrameric and dimeric PKM2 in chondrocytes treated with vehicle, DASA-58 (2, 10, and 50 μM) or TEPP-46 (10 and 50 μM) for 48 h in the presence of IL-1β, in a dose-dependent manner. Data were presented as means ± s.e.m., *n* = 6, one-way ANOVA with Tukey’s multiple comparisons. **e** Representative IF images of PKM2 in chondrocytes treated with 50 μM TEPP-46 for 48 h. **f** Representative immunoblots and **g** densitometric quantification of ADAMTS5, MMP3, MMP13, and COL2A1 in IL-1β-stimulated chondrocytes treated with TEPP-46 (10 and 50 μM) for 48 h. Data were presented as means ± s.e.m., *n* = 6, one-way ANOVA with Tukey’s multiple comparisons. **h** Representative SO&FG staining and **i** quantification of cartilage area in mouse femur head stimulated ex vivo with IL-1β (10 ng mL⁻¹) and treated with 50 μM TEPP-46 for 7 days. Data were presented as means ± s.e.m., *n* = 6, one-way ANOVA with Tukey’s multiple comparisons. *P < 0.05, **P < 0.01. ns not significant.

Genetic deletion and pharmacological inhibition by shikonin of PKM2 exhibited opposing effects on cartilage matrix metabolism, indicating that PKM2 may regulate matrix metabolism independently of its glycolytic enzyme activity. PKM2 exists in both tetrameric and dimeric conformations [32]. We therefore hypothesized that the tetramer–dimer balance of PKM2 may modulate ECM metabolism in OA. To this end, chemical cross-linking was performed to stabilize polymeric PKM2 species in human OA cartilage. Compared to non-OA cartilage, OA cartilage exhibited a clear increase in dimeric PKM2, accompanied by a marked reduction of its tetrameric form (Fig. 4a, b). Treatment of IL-1β–stimulated mouse chondrocytes with TEPP-46 or DASA-58, two small-molecule stabilizers of PKM2 tetramers [33], decreased dimer formation in a dose-dependent manner (Fig. 4c, d). TEPP-46 (50 μM) induced punctate and aggregated PKM2 signals in both the cytoplasm and nucleus (Fig. 4e). Similar to PKM2 knockdown, TEPP-46 notably reduced MMP3, MMP13, and ADAMTS5 expression and enhanced COL2A1 expression (Fig. 4f, g). Moreover, TEPP-46 reversed IL-1β-induced suppression of COL2A1 and induction of MMP3, MMP13, and ADAMTS5 in chondrocytes derived from PKM2^fl/fl^ mice (Supplementary Fig. 9a, b). However, no additive inhibitory effect on catabolic enzymes or additive stimulatory effect on COL2A1 was observed in Pkm2^icKO^ chondrocytes (Supplementary Fig. 9a, b), indicating that the effects of TEPP-46 are PKM2-dependent. Notably, prolonged TEPP-46 treatment for seven days alleviated IL-1β–induced cartilage destruction in murine femoral head explants (Fig. 4h, i).

**Fig. 5:**
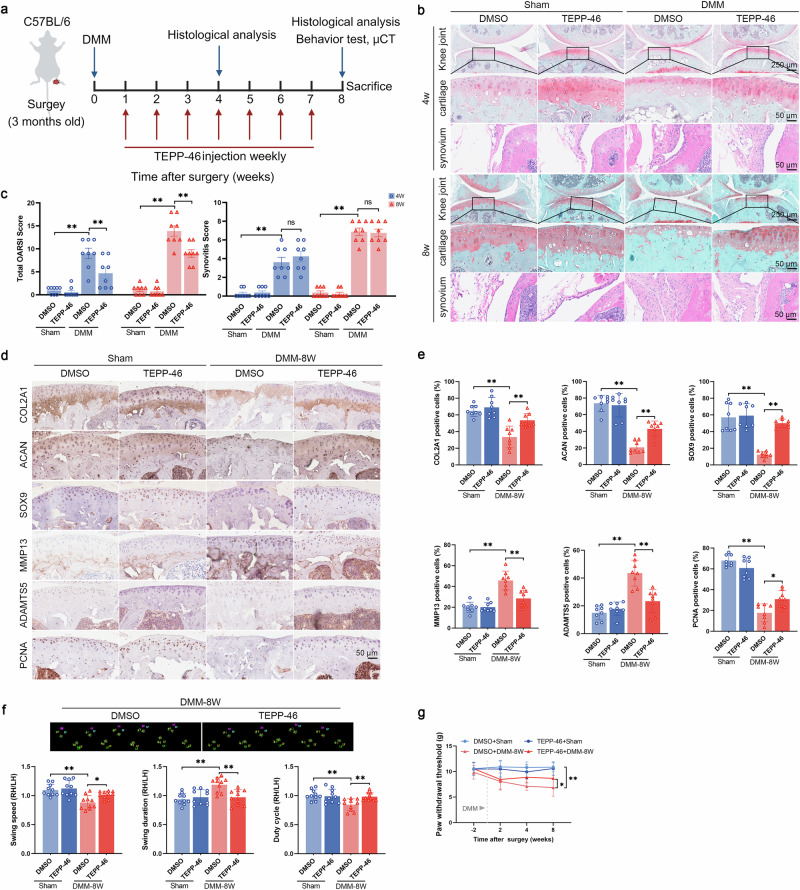
TEPP-46 treatment alleviates cartilage degeneration. **a** Schematic diagram of intra-articular TEPP-46 administration in DMM mice. Three-month-old mice underwent DMM surgery, and intra-articular injections of DMSO or TEPP-46 were initiated one week later and administered once weekly for seven weeks. Mice were euthanized at 4 or 8 weeks post-DMM for cartilage histology, hind-limb behavioral testing, and subchondral bone micro-CT. **b** Representative SO&FG staining of cartilages (top) and HE staining of synovium (bottom) in mice treated with TEPP-46, 4 weeks (upper panel) and 8 weeks (lower panel) post-DMM surgery. **c** Quantification of total OARSI scores (left) for cartilage and synovitis scores (right) in mice administered with DMSO or TEPP-46 at 4 weeks and 8 weeks post-DMM. **d** Representative IHC images and **e** quantification of COL2A1, ACAN, SOX9, MMP13, ADAMTS5, and PCNA expression in cartilage from mice treated with DMSO or TEPP-46, 8 weeks post-DMM surgery. **f** CatWalk gait analysis showing representative paw-print traces and quantification of hind-limb movement parameters (swing speed, swing duration, duty cycle), presented as RH/LH ratios. **g** Paw withdrawal threshold measured by the von Frey test. Data were presented as means ± s.e.m., *n* = 8, one-way ANOVA with Tukey’s multiple comparisons. *P < 0.05, **P < 0.01. ns not significant.

To evaluate the role of PKM2 tetramer stabilization in vivo, TEPP-46 was delivered intra-articularly following DMM surgery (Fig. 5a). No pathological abnormalities were detected in the heart, liver, lung, spleen, or kidneys of the mice (Supplementary Fig. 9c), supporting the safety of intra-articular treatment. TEPP-46 significantly reduced DMM-induced cartilage erosion but did not alter synovitis severity (Fig. 5b, c). TEPP-46 markedly increased COL2A1, ACAN, SOX9, and PCNA levels and reduced MMP13 and ADAMTS5 expression (Fig. 5d, e). Additionally, TEPP-46 further attenuated subchondral bone sclerosis 8 weeks post-surgery (Supplementary Fig. 10a, b). In both CatWalk and Von Frey analyses, TEPP-46 alleviated mechanical hypersensitivity and improved gait parameters (Fig. 5f, g and Supplementary Fig. 10c, d).

### PKM2 deficiency and tetramerization safeguard mitochondrial function

**Fig. 6:**
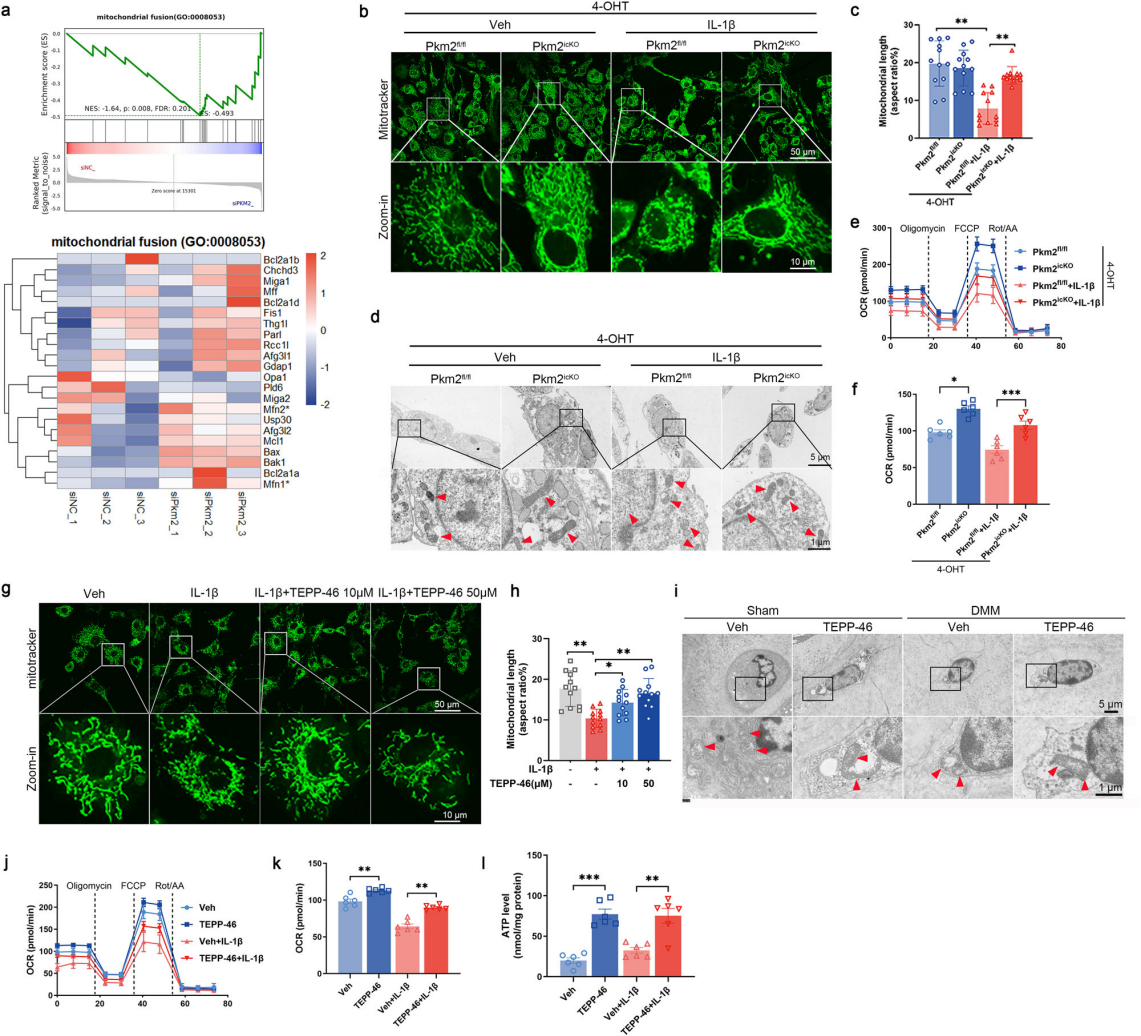
PKM2 deficiency and tetramerization safeguard mitochondrial function. **a** Gene-set enrichment analysis (GSEA) and corresponding gene-set heatmap illustrating mitochondrial fusion in chondrocytes transfected with siPkm2. **b** Representative MitoTracker staining (left) and **c** quantification of mitochondrial length (right) in IL-1β-stimulated chondrocytes isolated from Pkm2^fl/fl^ and Pkm2^icKO^ mice in the presence of 4-OHT. Data were presented as means ± s.e.m. from three independent experiments (*n* = 3), with four randomly selected cells per sample, one-way ANOVA with Tukey’s multiple comparisons. **d** Mitochondrial morphology in IL-1β-stimulated chondrocytes isolated from Pkm2^fl/fl^ and Pkm2^icKO^ mice in the presence of 4-OHT, visualized by transmission electron microscopy (TEM). **e** Real-time measurement of oxygen consumption rate (OCR) and **f** basal OCR quantification using the Seahorse assay in IL-1β–stimulated chondrocytes isolated from Pkm2^fl/fl^ and Pkm2^icKO^ mice in the presence of 4-OHT. FCCP, carbonyl cyanide p-trifluoromethoxyphenylhydrazone. Rot/AA, rotenone/antimycin A. **g** MitoTracker staining and **h** quantification of mitochondrial length (right) in chondrocytes incubated with vehicle or TEPP-46 (10, 50 μM). Data were presented as means ± s.e.m. from three independent experiments (*n* = 3), with four randomly selected cells per sample, one-way ANOVA with Tukey’s multiple comparisons. **i** Representative TEM images of mitochondria in chondrocytes from cartilage tissue of mice treated with vehicle or TEPP-46, collected eight weeks post-DMM surgery. **j** Real-time OCR measurement and **k** quantification of basal OCR in IL-1β–stimulated chondrocytes treated with vehicle or TEPP-46. Data were presented as means ± s.e.m., *n* = 6, one-way ANOVA with Tukey’s comparisons. **l** ATP levels measured in IL-1β-stimulated chondrocytes treated with vehicle or TEPP-46. Data were presented as means ± s.e.m., *n* = 6, one-way ANOVA with Tukey’s comparisons. *P < 0.05, **P < 0.01, ***P < 0.001. ns not significant.

Our previous work demonstrated that Pkm2 knockdown enhanced oxidative metabolism by upregulating tricarboxylic-acid–related genes and promoting oxygen consumption and ATP production [15], highlighting PKM2 as a critical regulator of mitochondrial respiration. Reanalysis of RNA-seq datasets (GSE281389) from Pkm2-knockdown primary mouse chondrocytes [15] revealed strong enrichment for mitochondrial fusion and oxidative phosphorylation modules (Fig. 6a and Supplementary Fig. 11a). To investigate whether Pkm2 deficiency affects mitochondrial dynamics, primary chondrocytes from adult Pkm2^fl/fl^ and Pkm2^icKO^ mice were treated with 4-hydroxytamoxifen (4-OHT) (Supplementary Fig. 11b, c). IL-1β stimulation induced fragmented mitochondria exhibiting punctate morphology (Fig. 6b, c). In contrast, PKM2-deficient cells displayed elongated tubular mitochondrial networks under the same conditions (Fig. 6b, c). Transmission electron microscopy (TEM) further confirmed altered mitochondrial morphology in Pkm2-deleted chondrocytes (Fig. 6d). To assess mitochondrial respiration, Seahorse assays were performed. Pkm2 deletion significantly elevated oxygen consumption rates under both basal and IL-1β-stimulated conditions (Fig. 6e, f), indicating enhanced oxidative phosphorylation. IL-1β exposure induced mitochondrial membrane depolarization and increased mitochondrial ROS (Supplementary Fig. 11d–g), and reduced the expression of mitochondrial biogenesis regulators peroxisome proliferator-activated receptor gamma coactivator 1-alpha (PGC-1α), nuclear respiratory factor 1 (NRF1), and mitochondrial transcription factor A (TFAM), which are central regulators of mitochondrial biogenesis (Supplementary Fig. 11h, i). Pkm2 deficiency restored mitochondrial membrane potential, reduced oxidative stress, and rescued mRNA and protein expression of PGC-1α, NRF1, and TFAM (Supplementary Fig. 11h–j). Consistent with Pkm2 depletion, TEPP-46 treatment ameliorated mitochondrial morphological disruption in vitro and in vivo, restored oxidative metabolism, and increased mitochondrial biogenesis proteins under IL-1β challenge (Fig. 6g–i and Supplementary Fig. 11k, l). Furthermore, TEPP-46 treatment significantly elevated oxygen consumption rates and intracellular ATP levels in chondrocytes under both basal and IL-1β-stimulated conditions (Figure 6j–l).Together, these findings demonstrate that Pkm2 deficiency and tetramer stabilization preserve mitochondrial function.

### MFN1-mediated mitochondrial fusion participates in maintaining cartilage homeostasis in Pkm2-deficient conditions

**Fig. 7:**
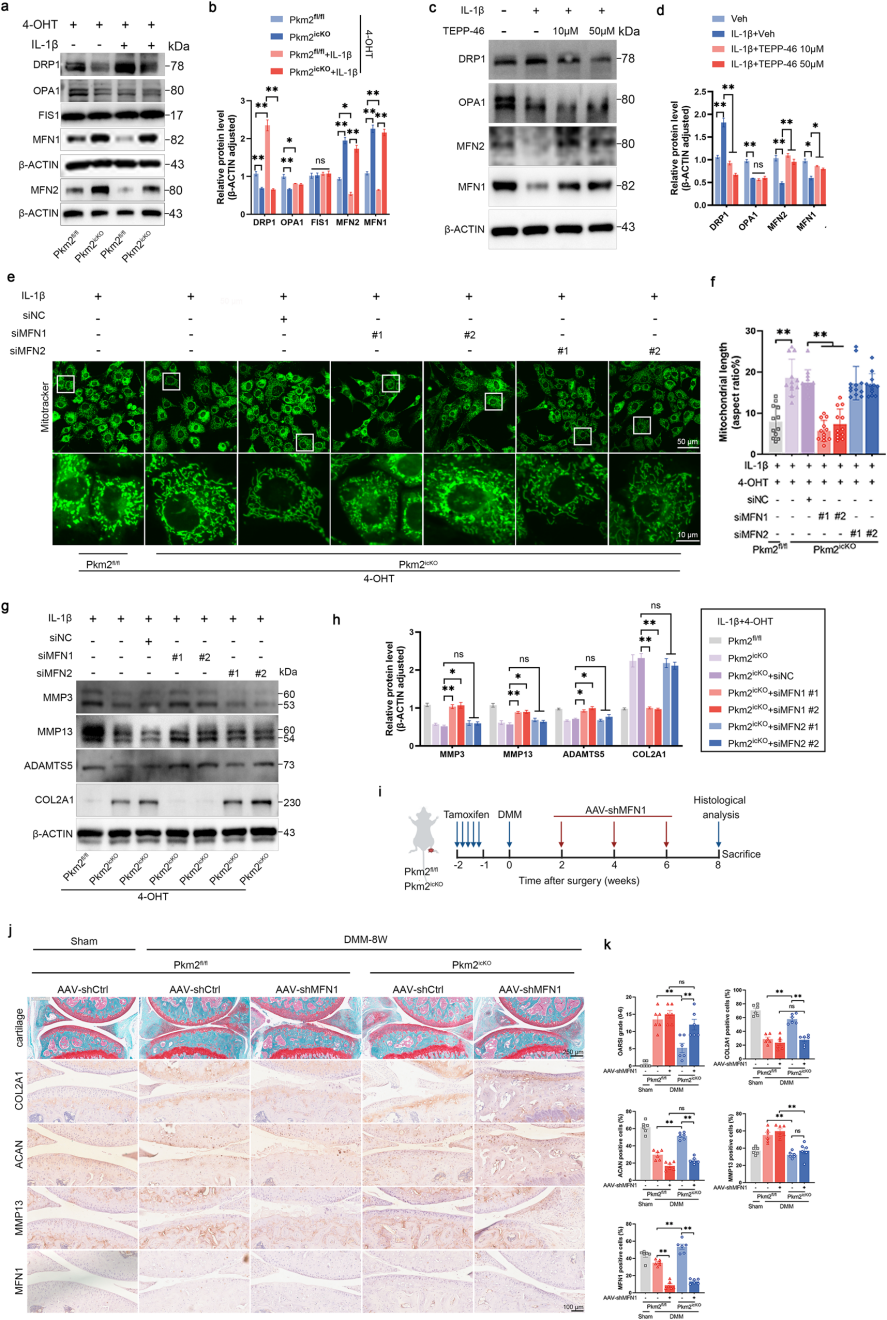
MFN1, not MFN2, mediates mitochondrial fusion and ECM homeostasis upon PKM2 deficiency or tetramerization. **a** Representative immunoblots and **b** densitometric quantification of DRP1, OPA1, FIS1, MFN1, and MFN2 in IL-1β-stimulated chondrocytes isolated from Pkm2^fl/fl^ and Pkm2^icKO^ mice in the presence of 4-OHT. Data were presented as means ± s.e.m., *n* = 6, one-way ANOVA with Tukey’s multiple comparisons. **c** Representative immunoblots and **d** densitometric quantification of DRP1, OPA1, MFN1, and MFN2 in IL-1β-stimulated chondrocytes incubated with vehicle or TEPP-46 (10, 50 μM). Data were presented as means ± s.e.m., *n* = 6, one-way ANOVA with Tukey’s multiple comparisons. **e** Representative mitotracker staining and **f** quantification of mitochondrial length in Pkm2^fl/fl^ and Pkm2^icKO^ chondrocytes transfected with siNC, siMfn1 or siMfn2 in the presence of 4-OHT and IL-1β. Data were presented as means ± s.e.m. from three independent experiments (*n* = 3), with four randomly selected cells per sample, one-way ANOVA with Tukey’s multiple comparisons. **g** Representative immunoblots and **h** densitometric quantification of MMP3, MMP13, ADAMTS5, and COL2A1 in Pkm2^fl/fl^ and Pkm2^icKO^ chondrocytes transfected with siNC, siMfn1 or siMfn2 in the presence of 4-OHT and IL-1β. Data were presented as means ± s.e.m., *n* = 6, one-way ANOVA with Tukey’s multiple comparisons. **i** Schematic diagram of intra-articular AAV-shMFN1 administration in cartilage-specific PKM2 deletion mice subjected to DMM surgery. Three-month-old Pkm2^fl/fl^ and Pkm2^icKO^ mice received tamoxifen intraperitoneally for 5 consecutive days, followed by DMM surgery. Intra-articular injections of AAV-shMFN1 were initiated 2 weeks after DMM surgery and administered every 2 weeks for a total of three doses. Cartilage was harvested for histological analysis 8 weeks post-DMM. **j** Representative SO&FG and IHC staining images of COL2A1, ACAN, MMP13, and MFN1 in the cartilage tissues from Pkm2^fl/fl^ and Pkm2^icKO^ mice treated with AAV-shCtrl and AAV-shMFN1. **k** Quantification of OARSI scores and IHC signals for COL2A1, ACAN, MMP13, and MFN1. Data were presented as means ± s.e.m., *n* = 6, one-way ANOVA with Tukey’s multiple comparisons. *P < 0.05, **P < 0.01. ns not significant.

Mitochondrial morphology and bioenergetic capacity are tightly controlled by a conserved network of fusion- and fission-promoting GTPases [34]. In mammalian cells, outer-membrane fusion is mediated by MFN1 and MFN2, whereas inner-membrane fusion is executed by OPA1 [35]. Conversely, mitochondrial fission is driven by DRP1, which is recruited from the cytosol to mitochondria via adapters including mitochondrial fission 1 protein (FIS1), mitochondrial fission factor (MFF) or mitochondrial dynamics protein of 49/51 kDa (MiD49/51) [36]. We next assessed the expression of mitochondrial dynamics-related proteins in Pkm2^icKO^ chondrocytes under IL-1β stimulation. Pkm2 deletion increased MFN1 and MFN2 expression while reducing DRP1 levels, with no detectable changes in OPA1 or FIS1 (Fig. 7a, b). Although DRP1 upregulation has been reported to drive mitochondrial fission, our observations did not align with this pattern. We therefore focused on the fusion regulators MFN1 and MFN2. Consistently, TEPP-46 treatment also upregulated MFN1 and MFN2 in IL-1β-stimulated chondrocytes (Fig. 7c, d).

To determine the specific roles of MFN1 and MFN2, we performed siRNA-mediated knockdown (Supplementary Fig. 12). Knockdown of Mfn1, but not Mfn2, induced fragmented and punctate mitochondrial morphology in IL-1β-stimulated Pkm2^icKO^ chondrocytes (Fig. 7e, f). Furthermore, knockdown of Mfn1, but not Mfn2, reversed the protective effects of Pkm2 deficiency on ECM protein expression, restoring IL-1β-induced changes in COL2A1, MMP3, MMP13, and ADAMTS5 levels (Fig. 7g, h), implying a key role of MFN1 in governing mitochondrial fusion and extracellular matrix homeostasis in the absence of Pkm2. Consistent with these findings, MFN1 protein expression was reduced in human OA cartilage and surgery-induced mouse OA cartilage (Supplementary Fig. 13).

To further investigate the role of MFN1 in Pkm2-deficient chondrocytes in vivo, AAV-shMFN1 was administered via intra-articular injection in Pkm2^icKO^ mice subjected to DMM surgery (Fig. 7i). AAV-shMFN1 administration exacerbated cartilage damage and ECM degradation in Pkm2^fl/fl^ mice subjected to DMM surgery, and abolished the protective effects observed in Pkm2^icKO^ mice when compared with the AAV-shCtrl administration (Fig. 7j, k). Collectively, these findings demonstrate that MFN1 plays a central role in maintaining mitochondrial fusion and extracellular matrix homeostasis in Pkm2-deficient chondrocytes.

### PKM2/ERK axis regulates MFN1 upregulation in chondrocytes

**Fig. 8:**
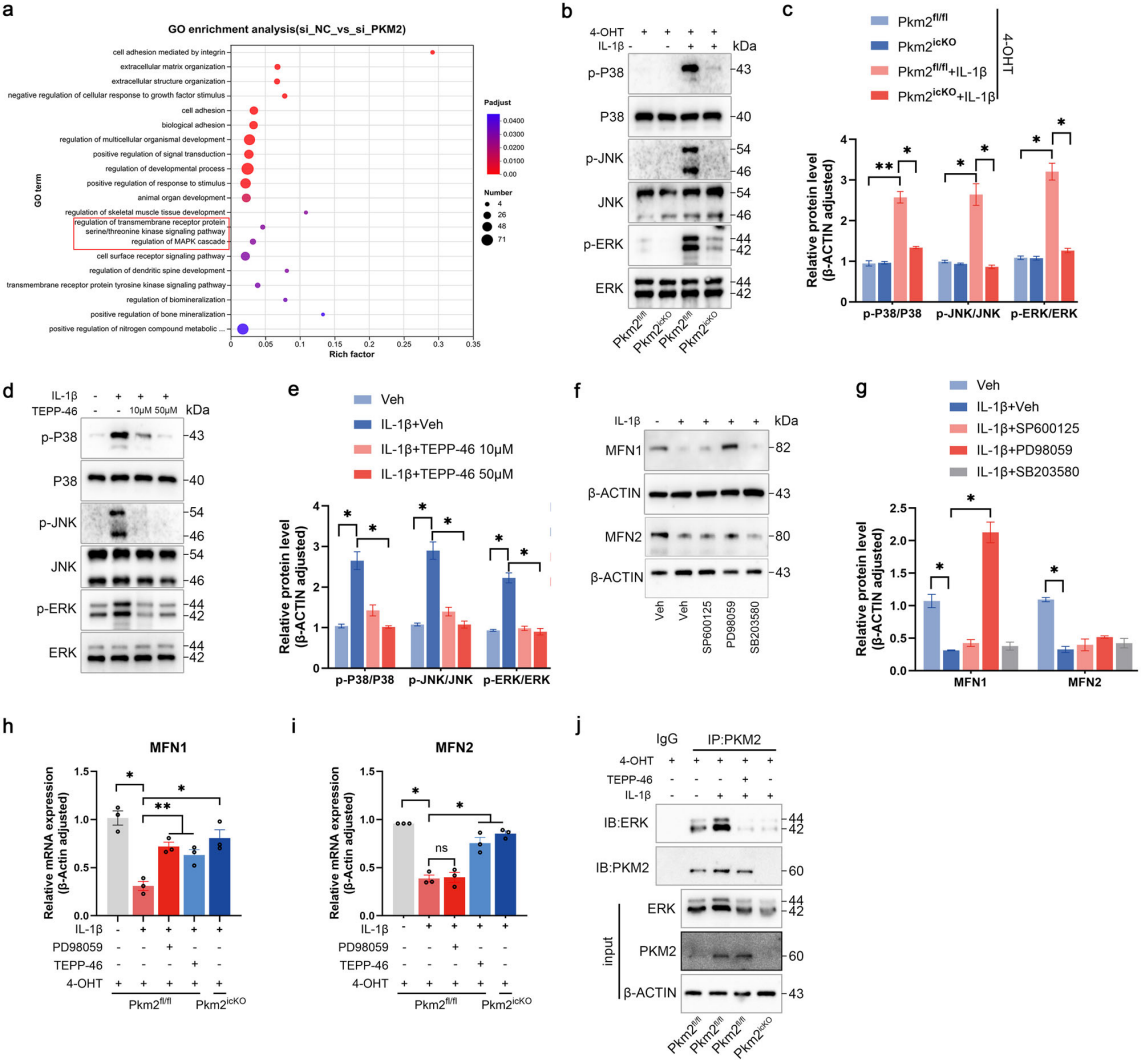
PKM2–ERK interaction regulates MFN1 expression. **a** GO enrichment analysis of chondrocytes transfected with siPkm2. **b** Representative immunoblots and **c** densitometric quantification of MAPKs in IL-1β-stimulated chondrocytes isolated from Pkm2^fl/fl^ and Pkm2^icKO^ mice in the presence of 4-OHT. **d** Representative immunoblots and **e** densitometric quantification in IL-1β-stimulated chondrocytes treated with vehicle or TEPP-46 (10 and 50 μM). **f** Representative immunoblots and **g** densitometric quantification of MFN1 and MFN2 in IL-1β-stimulated chondrocytes incubated with JNK inhibitor SP600125 (20 μM), p38 inhibitor SB203580 (10 μM) or ERK inhibitor PD98059 (10 μM). **h** Relative MFN1 and **i** MFN2 mRNA expression in IL-1β-stimulated Pkm2^fl/fl^ and Pkm2^icKO^ chondrocytes treated with vehicle, TEPP-46 (50 μM) or PD98059 (10 μM) in the presence of 4-OHT. **j** Representative Co-IP immunoblots of PKM2 and ERK in IL-1β-stimulated Pkm2^fl/fl^ and Pkm2^icKO^ chondrocytes treated with vehicle or TEPP-46 (50 μM) in the presence of 4-OHT. IP Immunoprecipitation, IB Immunoblotting. Data were presented as means ± s.e.m., *n* = 3, one-way ANOVA with Dunnett’s comparisons. *P < 0.05, **P < 0.01.

To elucidate the mechanism by which Pkm2 deficiency or TEPP-46 treatment induces MFN1 upregulation, endogenous co-immunoprecipitation (co-IP) was first performed to assess a potential association between PKM2 and MFN1. No direct interaction was detected (data not shown), consistent with immunofluorescence staining that demonstrated limited spatial colocalization of PKM2 and MFN1 in murine cartilage (Supplementary Fig. 13a). To identify the potential signaling pathways, transcriptomic profiles of Pkm2-knockdown chondrocytes (GSE281389) were analyzed. Gene ontology (GO) enrichment analysis revealed significant involvement of transmembrane receptor regulation, protein serine/threonine kinase signaling, and MAPK cascade regulation (Fig. 8a). Functionally, IL-1β stimulation markedly increased phosphorylation of p38, c-Jun N-terminal kinase (JNK), and extracellular signal-regulated kinase (ERK), normalized to total protein levels (Fig. 8b–e). Conversely, Pkm2 deletion or pharmacologic inhibition of Pkm2 dimerization suppressed MAPK activation (Fig. 8b–e). To dissect the specific MAPK mediating MFN1 regulation, IL-1β-stimulated chondrocytes were treated with selective inhibitors: SP600125 (JNK), SB203580 (p38), and PD98059 (ERK). While none of the inhibitors affected IL-1β-induced MFN2 downregulation, PD98059 partially restored MFN1 expression, indicating that ERK signaling specifically mediates MFN1 upregulation (Fig. 8f, g). In line with this, IL-1β–induced MFN1 downregulation, but not MFN2 suppression, was partially reversed by PD98059. Pkm2 deficiency or TEPP-46 treatment enhanced both MFN1 and MFN2 transcription (Fig. 8h, i). However, co-IP demonstrated that PKM2 directly interacts with ERK, and IL-1β stimulation further strengthened this interaction; conversely, TEPP-46 treatment or Pkm2 deletion attenuated it (Fig. 8j). Taken together, these findings identify PKM2 as a key modulator of ERK signaling–mediated MFN1 upregulation, while MFN2 expression is regulated through an ERK-independent pathway, highlighting distinct regulatory mechanisms governing mitochondrial fusion proteins.

To further investigate the correlation between *PKM* expression and cartilage matrix metabolism, we analyzed an additional scRNA-seq dataset (GSE104782, Supplementary Fig. 14a, b). Chondrocytes with the top 10% of *PKM* gene expression were classified as PKM^high^, while those in the bottom 10% were classified as PKM^low^. Comparative analysis revealed that PKM^low^ cells were enriched in gene modules associated with transmembrane receptor serine/threonine kinase signaling, extracellular structure organization, extracellular matrix organization, and cartilage development (Supplementary Fig. 14c, d). KEGG pathway analysis further highlighted enrichment in ECM–receptor interaction pathways (Supplementary Fig. 14d), linking PKM expression to cartilage matrix regulation.

## Discussion

The present study demonstrates that during OA progression, the glycolytic enzyme PKM2 undergoes a conformational shift from tetrameric to dimeric forms in chondrocytes. This allosteric transition alters PKM2–ERK interactions, leading to downregulation of the mitochondrial fusion protein MFN1, mitochondrial dysfunction, and subsequent disruption of extracellular matrix homeostasis.

Due to their distinctive anatomical location, chondrocytes primarily rely on glycolysis for ATP production [37]. In the present study, the glycolytic rate-limiting enzyme PKM2 is upregulated in patients and animal models of osteoarthritis, implying that enhanced glycolysis occurs in osteoarthritis [5, 38]. Notably, the presence of PKM2 is significantly increased, while PKM1 is barely detected. Both PKM1 and PKM2 proteins have been identified in cultured human chondrocytes [11]. Furthermore, a moderate increase in PKM1 expression in four-week-old Pkm2^icKO^ mice suggests that alternative splicing of the PKM pre-mRNA has a compensatory role during the developmental stage [39–42]. PKM2 protein exists as both allosteric dimers and tetramers, which are composed of identical monomers [43]. The dimers take part in transcriptional regulation, whereas the tetramers favor glycolysis. The decreased tetramer-to-dimer ratio of PKM2 in OA cartilage supports the hypothesis that energy metabolism has been switched in the disease. Deletion of PKM2, either by siPkm2 or chondrocyte-specific PKM2 knockout, restores ECM homeostasis and slows the progression of OA. Moreover, TEPP-46, an activator of PKM2 that promotes tetramerization of PKM2 oligomers [9, 16, 44, 45], exerts protective effects similar to PKM2 deletion. In fact, TEPP-46 stabilizes PKM2 in its tetrameric form and prevents its nuclear translocation, thereby restricting its non-metabolic signaling functions without mimicking a complete loss of PKM2. Although the mechanisms differ, several studies have shown that TEPP-46 treatment and PKM2 deficiency can lead to comparable phenotypic outcomes when nuclear PKM2 signaling is the dominant driver. TEPP-46 or PKM2 knockout similarly suppresses Th17 differentiation in T cells [46], and both interventions reduce inflammatory injury in myeloid-driven disease models [47]. These data support our observation that limiting PKM2 nuclear activity, either genetically or pharmacologically, can converge on similar biological effects.

Four major metabolic processes, including glycolysis, oxidative phosphorylation, fatty acid oxidation, and glutamine metabolism, are dynamically interchangeable in response to physio-pathological stimuli. Both bioinformatic analyses and data in primary cultured chondrocytes in the present study, including changes in protein levels related to mitochondrial dynamics and biogenesis, as well as increased mitochondria-derived oxidative stress, indicate that mitochondrial dysfunction follows PKM2 dimerization. Mitochondria play key roles in ATP production, reactive oxygen species generation, and cell apoptosis [48, 49]. In this study, increased DRP1 protein and decreased MFN1 and MFN2 proteins confirm that mitochondrial dynamics are biased towards fission, resulting in mitochondrial fragmentation, elevated oxidative stress [23, 50–55], increased apoptosis, and further leading to ECM degradation in cultured chondrocytes [27, 56, 57]. MFN1 and MFN2 share 81% sequence identity [58, 59]. The former is localized in the outer mitochondrial membrane [60], while the latter is distributed in the outer mitochondrial, endoplasmic reticulum membranes, and peroxisome [61]. These two mitofusins coordinate in embryonic development [58] and glucose-stimulated insulin secretion in β-cells [62], though they are not essential for sperm development [63]. MFN1 and MFN2 form three distinct molecular complexes: MFN1 homotypic oligomers, MFN2 homotypic oligomers, and MFN1-MFN2 heterotypic oligomers, to promote mitochondrial fusion [63]. Notably, the MFN1-MFN2 heterotypic oligomers can compensate for mutant MFN2 in Charcot-Marie-Tooth disease type 2 A [64]. In this study, inhibiting MFN1, but not MFN2, abolishes the protective effects in chondrocyte-specific PKM2-deleted mice subjected to DMM surgery, further emphasizing the distinctive role of MFN1 in mitochondria as well as mitochondrial dynamic regulation [60].

Metabolic processes respond quickly to environmental stress and pathological stimuli [19]. Thus, it is reasonable to hypothesize that PKM2 interacts physically with mitochondria or MFN proteins, as dimeric PKM2 has the potential to translocate to mitochondria [17]. It has been reported that the expression of PKM2 is positively correlated to MFN2 protein levels in human breast carcinoma MCF-7 cells [65] through interactions between the N-terminus of PKM2 and the C-terminus of MFN2 [52]. In the present study, mitochondrial MFN1, but not MFN2, responds oppositely to the dimeric PKM2. Combined with the lack of colocalization between PKM2 and MFN1, these findings suggest that PKM2 regulates MFN1 expression indirectly rather than through a direct protein–protein interaction. The MAPK signaling pathway plays a key role in regulating cell proliferation, differentiation, and apoptosis [66–68]. It has been reported that ERK inhibits MFN1 function by phosphorylating Thr562 in primary cortical neurons [69]. In this study, the MFN1 protein expression is restored by ERK inhibition, supporting that activation of ERK signaling pathways plays a critical role in governing mitochondrial function [70, 71].

The nuclear presence of PKM2 observed in the present study suggests that PKM2 translocates to the nucleus, where it participates in protein regulation [8]. Nuclear PKM2 functions as a transcriptional regulator through interactions with multiple transcription factors, including HIF-1α/2α [72, 73], p53 [73], β-catenin [74], and octamer-binding transcription factor 4 [75]. In addition, PKM2 has been reported to associate with the endothelial nitric oxide synthase complex under basal conditions, with endothelial nitric oxide synthase exerting intrinsic inhibitory effects on PKM2 activity through nitrosation [76]. Taken together with our observation that dimeric PKM2 binds to and activates ERK kinase, these findings indicate that PKM2 exerts multifaceted, non-glycolytic functions [77]. Therefore, the downregulation of MFN1 observed in this study is likely mediated by both direct and indirect regulatory mechanisms affecting protein expression, which warrant further investigation.

The involvement of PKM2 in cellular senescence has been studied in recent years [15, 78] [34, 80]. In the present study, the comparable presence of p16^INK4a^ and p21 in DMM-induced osteoarthritis and aging mice confirms that aging, or premature aging, is a crucial risk factor for the disease. In line with this, PKM2 protein aggregation has also been reported in senescent cells and tissues of aged mice, and dissolving this protein aggregation decelerates the progression of aging and extends lifespan in aged mice [79].

In summary, dimeric PKM2 inhibits mitochondrial fusion in chondrocytes, disrupting extracellular matrix homeostasis. Reduced expression of dimeric PKM2 mitigates DMM-induced cartilage damage, alleviates joint pain, and decelerates the progression of OA. Given that knee arthroplasty is the current medical procedure for patients with severe osteoarthritis, targeting dimeric PKM2 offers new insights into the therapeutic strategy of osteoarthritis.

## Materials and methods

### Reagents

Recombinant mouse interleukin (IL)-1β was obtained from R&D Systems (#401-ML-010, Minneapolis, USA). TEPP-46 (#S7302) and DASA-58 (#S7928) were purchased from Selleck Chemicals (Houston, TX, USA). Shikonin was sourced from MedChemExpress (#HY-N0822, Monmouth Junction, NJ, USA). SP600125 (#1496), SB203580 (#1202), and PD98059 (#1213) were purchased from Tocris (Bristol, UK).

### DSS cross-linking assay for PKM2 oligomers

A DSS cross-linking assay was conducted using a protein cross-linking agent disuccinimidyl suberate (DSS, #21555, Thermo Scientific), to evaluate the formation of PKM2 monomers, dimers, and tetramers. Cultured chondrocytes were incubated with 5 nM DSS for 2 h on ice. The cross-linking reaction was terminated by 20 mM Tris solution. Of note, human cartilage tissue was incubated with 500 μM DSS for 2 h. The protein samples from both cartilage tissue and chondrocytes were loaded with native gel sample loading buffer (Beyotime) without boiling, then separated using 6% PAGE gel.

### MitoTracker staining for mitochondrial labeling

Mitochondria were labeled using MitoTracker Green FM (#40742ES50, Yeasen). In brief, cultured chondrocytes were incubated with 500 nM MitoTracker in PBS at 37 °C for 45 min. After PBS washes, the mitochondrial morphology was captured using a confocal laser scanning microscope (Olympus) with an excitation wavelength of 490 nm. Mitochondrial length was quantified using ImageJ software.

### Mitochondrial stress test (OCR measurement)

Cellular oxygen consumption was assessed using a mitochondrial stress test kit following the manufacturer’s instructions. Briefly, chondrocytes were seeded in Seahorse XF microplates and equilibrated in assay medium prior to measurement. Basal respiration and sequential responses to oligomycin, FCCP, and a respiratory chain inhibitor mixture were recorded. OCR values were normalized to cell number and analyzed using the accompanying software.

### Measurement of intracellular ATP

Intracellular ATP levels were quantified using a commercial ATP detection kit (Beyotime). Following 24 h stimulation with IL-1β (10 ng mL⁻¹), cells were lysed, and the supernatants were subjected to luminescence-based ATP measurement using a microplate luminometer (Thermo Fisher Scientific). ATP content was normalized to total protein in each sample and reported as nmol per mg protein.

### Co-immunoprecipitation

Chondrocytes were lysed using the IP lysis buffer supplied with the rProtein A/G Magnetic IP/Co-IP Kit (Absin) supplemented with protease inhibitors. Lysates were incubated with either the target antibody or control IgG and rProtein A/G magnetic beads at 4 °C for 2–4 h. After washing away unbound proteins, the immunoprecipitated complexes were eluted and subjected to SDS-PAGE and immunoblotting with the indicated antibodies.

### CatWalk gait analysis and Von Frey tests

Mechanical sensitivity of the right hind paw after the surgery was evaluated using CatWalk gait analysis and Von Frey tests. Gait analysis was conducted to evaluate locomotor function and coordination by using the CatWalk XT system (Noldus, Netherlands). Mouse movements on a glass walkway, including paw prints, stride length, stance duration, and swing phase, were recorded. In the test, each animal completed a minimum of three valid runs. Data were analyzed using CatWalk XT software. For the Von Frey test, mice were acclimated in a transparent chamber with a mesh platform for 30 min. Calibrated Von Frey filaments (0.008 g to 2.0 g, Stoelting, USA) were then applied perpendicularly to the plantar surface of the hind paw. Each filament was applied for about 2 s, with a 10-s interval. The 50% withdrawal threshold was determined using the up-down method, with paw withdrawal or licking considered a positive response.

### Intra-articular Injections

DMM surgery was performed on the right knee of the mice, when they were 3 months old. Treatment with the intra-articular injection was conducted 1 week after the DMM surgery. The knee joint cavity beneath the patella was injected with adeno-associated virus (AAV), small interfering RNA (siRNA), or TEPP-46.

Recombinant adeno-associated virus vectors designed for Mfn1 knockdown (AAV-shMfn1), PKM2 overexpression (AAV-PKM2), and the corresponding negative control (AAV-shCtrl) were obtained from GenePharma (Shanghai, China). The AAV-shCtrl or AAV-sh*Mfn1* was prepared in 10 μl (1.0 × 10¹² vg ml^−1^) and administered biweekly through intra-articular injection.

The siRNA targeting PKM2 (siPkm2 #3, Supplementary Table 2 and Supplementary Fig. 4b, c) was chemically modified with 2’Ome (GenePharma). siPkm2 (50 nM) was dissolved in phosphate-buffered saline (PBS) and delivered twice a week via intra-articular injection using a siRNA mate (GenePharma). TEPP-46 (50 μM) was dissolved in DMSO and administered weekly through intra-articular injection.

### Statistics and reproducibility

All statistical analyses were conducted using GraphPad Prism 8 software (San Diego, USA). Normality was assessed with the Shapiro–Wilk test, and homogeneity of variances was evaluated using Levene’s test. Student’s *t*-test was employed for comparisons between two groups, while ANOVA with post-hoc corrections was used for comparisons involving more than two groups. A *P* value of less than 0.05 was considered statistically significant. All experiments were performed independently in at least triplicate, and data were presented as means ± s.e.m. Graphical elements were created using BioRender.

## Supplementary information

Supplementary file

Original Western blots

aj-checklist.

## Data Availability

All data needed to evaluate the conclusions in the paper are present in the paper and/or the Supplementary Materials. The raw sequence data reported in this article have been deposited in the GEO under accession no. GSE281389.
