# Supplementary material for: Dimeric PKM2 in chondrocytes impairs mitochondrial homeostasis in osteoarthritis
Source: Cell Death Dis. 2026 Mar 25;17(1):370. doi: 10.1038/s41419-026-08621-4 (PMC13039331; doi:10.1038/s41419-026-08621-4)
Supplement: Supplementary file 1 — Supplementary file [file 41419_2026_8621_MOESM1_ESM.docx]

**Dimeric PKM2 in chondrocytes impairs** **mitochondrial** **homeostasis in osteoarthritis**

Bo Liu^1,^^†^, Yun Liang^1,†^, Chenzhong Wang^1,†^, Ziyu Weng^1^, Yi Yang^1^, Yi Shi^2,3^*, Chi Zhang^1,^*

^1^ Department of Orthopedic Surgery, Zhongshan Hospital, Fudan University, Shanghai, China.

^2^ Department of Kidney Transplantation, Zhongshan Hospital, Fudan University, Shanghai, China.

^3^ Shanghai Key Laboratory of Organ Transplantation, Zhongshan Hospital, Fudan University, Shanghai, China.

^†^BL, YL and CW contributed equally to this work.

**Corresponding Authors:**

Prof. Zhang, Chi

Department of Orthopedic Surgery, Zhongshan Hospital, Fudan University, Shanghai, China

E-mail: zhang.chi@zs-hospital.sh.cn

ORCID: 0009-0007-1639-3784

Dr. Shi, Yi

Department of Kidney Transplantation, Zhongshan Hospital, Fudan University, Shanghai, China

Shanghai Key Laboratory of Organ Transplantation, Zhongshan Hospital, Fudan University, Shanghai, China

E-mail: [shi.yi@zs-hospital.sh.cn](mailto:shi.yi@zs-hospital.sh.cn)

ORCID: 0000-0003-3005-9655

**This file includes:**

**Supplementary Materials and Methods**

**Supplementary Figures S1-S14**

**Supplementary Tables S1-S5**

**Supplementary Materials and Methods**

**Mice and Experimental Post-Traumatic Osteoarthritis**

The PKM2^flox/flox^ and Col2-Cre^ERT^ mice were generated by Cyagen Biosciences Inc. (Jiangsu, China) based on CRISPR/Cas9-mediated genome engineering. The *Pkm* gene, located on mouse chromosome 9, consists of twelve exons, with the ATG start codon in exon 2 and the TGA stop codon in exon 11 (Transcript Pkm-201: ENSMUST00000034834). For the conditional PKM2 deletion, exon 10, which contains a 167 bp coding sequence specific to the PKM2 isoform, was targeted. Chondrocyte-specific PKM2 knockout mice (Col2-Cre^ERT^; PKM2^flox/flox^) were generated by crossbreeding PKM2^flox/flox^ mice with Col2-Cre^ERT^ mice. Two-week-old and three-month-old male mice (PKM2^flox/flox^ and Col2-Cre^ERT^; PKM2^flox/flox^) were used in the present study. The two-week-old mice were used to confirm the gene mutation, which were euthanized after tamoxifen administration (Supplementary Figure 5). Mice received intraperitoneal injections of tamoxifen at a dose of 100 mg kg^-1^ (#T5648, 20 mg ml^-1^ dissolved in corn oil, Sigma-Aldrich, St. Louis, MO, USA) for five consecutive days. After the tamoxifen injection, three-month-old mice underwent destabilization of the medial meniscus (DMM) surgery [1]. Mice in the sham group underwent the same procedures but did not destroy the meniscus. Mice were sacrificed four and eight weeks after the surgery (Figure 3 and Figure 5). All animal procedures were conducted following the guidelines set by the National Institutes of Health Guide for the Care and Use of Laboratory Animals and were approved by the Ethics Committee for Animal Research of Zhongshan Hospital.

**Mouse** **Genotyping**

All mouse pups were genotyped by PCR examination. Briefly, tissue genomic DNA was combined with 2 × Taq Master Mix (Vazyme, Nanjing, China) and primers. The PCR process began with an initial denaturation at 94°C for three minutes, followed by 35 cycles consisting of denaturation at 94°C for 30 seconds, annealing at 60°C for 35 seconds, and extension at 72°C for 35 seconds, with a final extension at 72°C for five minutes. Agarose gel electrophoresis was performed, and the results were captured using the GelDoc Go (BIO-RAD, Hercules, USA). The primer sequences were listed in Supplementary Table 1.

**Small Interfering RNA (siRNA) Transfection**

Cultured chondrocytes (60% to 80% confluency) were transfected with small interfering RNAs (siRNAs) or their scrambled controls (GenePharma, RNA sequences listed in Supplementary Table 2). 50 nM siRNA was introduced into chondrocytes using Lipofectamine RNAiMAX (#13778150, Invitrogen, Carlsbad, USA) following the manufacturer's instructions. Knockdown efficiency was assessed 48 hours after transfection.

**Isolation, Culture, and Identification of Murine Chondrocytes**

Murine articular chondrocytes were isolated from the knee joints of 8-10-week-old mice. The articular cartilage was carefully separated from the tibial plateau and femoral condyles. Cartilage fragments were digested overnight at 37°C in DMEM/F12 (Gibco, Waltham, USA) containing 0.2% type II collagenase (Gibco) and 10% fetal bovine serum (FBS; Gibco). The digested tissue was filtered by a 70-μm strainer and rinsed with PBS. Primary mouse chondrocytes were cultured in DMEM/F12 medium containing 10% FBS at 37°C (Supplementary Figure 4a). The primary chondrocytes without passaging were used in the present study.

To induce the specific knockout of PKM2 in chondrocytes *in vitro*, primary chondrocytes isolated from PKM2^flox/flox^ and Col2-Cre^ERT^; PKM2^flox/flox^ mice were incubated with 1 μM 4-hydroxytamoxifen (4-OHT, #H7904, Sigma-Aldrich) for 24 hours.

In cultured experiments, cells were stimulated with IL-1β (10 ng ml^-1^) for 24 hours. TEPP-46 (10 μM or 50 μM), DASA-58 (2 μM, 10 μM or 50 μM), Shikonin (1 μM) were incubated for 48 hours prior to IL-1β stimulation. SP600125 (20 μM), PD98059 (10 μM), and SB203580 (10 μM) were incubated for two hours prior to IL-1β stimulation.

**Clinical Samples**

Osteoarthritis articular cartilage was collected from patients who had undergone total knee arthroplasty (n=6), while control articular cartilage samples were obtained from patients who had undergone lower limb amputation due to tumor lesions or trauma (non-articular regions) (n=6) at Zhongshan Hospital, Fudan University, Shanghai, China. The clinical characteristics of the patients are provided in Supplementary Table 3. Informed consent for donating biological samples was acquired from the patients, and the study received approval from the Ethics Committee of Zhongshan Hospital.

**RNA Extraction and Quantitative Real-Time PCR**

Total RNA was extracted from primary chondrocytes using TRIzol reagent (Sigma-Aldrich). The RNA purity and concentration were determined using a NanoDrop spectrophotometer (Thermo Fisher Scientific, Waltham, MA, USA). Reverse transcription of mRNA to complementary DNA was conducted by the First Strand cDNA synthesis kit (Yeasen, Shanghai, China). Real-time qPCR was conducted using Hieff qPCR SYBR Green Master Mix (Yeasen, Shanghai, China) and detected by ABI Quant Studio 7 Flex (Applied Biosystems, Foster City, CA, USA). Target mRNA expression levels were normalized to that of *β-Actin* and calculated using the 2^-ΔΔC^_T_ method. The primer sequences are detailed in Supplementary Table 4.

**Histological Analysis, Scoring System, Immunofluorescence, and Immunohistochemistry**

Human cartilage and mouse knee joint tissues were fixed in 4% paraformaldehyde (Servicebio, Wuhan, China), decalcified in 10% EDTA solution (Yeasen, Shanghai, China), dehydrated, and then embedded in paraffin. Serial sections (6 μm-thick) were collected for histological staining. For hematoxylin and eosin (HE) staining, sections were first stained with hematoxylin for three minutes, followed by eosin staining for two minutes. For SO&FG staining, sections were stained with 0.1% Safranin O solution (#TMS-009, Sigma-Aldrich) and 0.001% Fast Green solution (#F7252, Sigma-Aldrich). The Mankin histological grading system was employed to evaluate the severity of cartilage erosion in human samples [2].

Cartilage damage was evaluated using the OARSI scoring system. Four images from the medial and lateral femoral condyle and tibial plateau of each mouse were blindly evaluated by a technician. The final OARSI score was determined by summing the average femoral and tibial scores from the four images [3]. Synovitis was scored as described in previous studies [4].

For immunofluorescence staining, the sections underwent antigen retrieval using microwave heating in a pH 8.0 EDTA solution, followed by permeabilization with 0.2% Triton X-100 (Yeasen, Shanghai, China). After blocking with goat serum (Beyotime, Shanghai, China) for one hour, the sections were incubated with primary antibodies (details provided in Supplementary Table 5) overnight at 4°C. Following washing, the sections were incubated with fluorescence-conjugated secondary antibodies for one hour and followed by staining with DAPI for 5 minutes. Images were obtained using a confocal laser scanning microscope (Olympus, Tokyo, Japan). For immunohistochemistry examination, the sections were first incubated with an H_2_O_2_ blocking agent (Gene Tech, Shanghai, China) for 10 minutes. After antigen retrieval, the sections were incubated with primary antibodies, secondary antibodies, and stained using DAB Detection Kit (#GK600510, Gene Tech). Images were obtained using a Digital Pathology Scanner (Leica, Wetzlar, Germany). The percentage of positive cells was quantified by ImageJ software.

**TUNEL Staining for Apoptosis Detection**

Chondrocyte apoptosis levels were evaluated using the TdT-mediated dUTP Nick-End Labeling (TUNEL) Cell Apoptosis Detection Kit (Beyotime). Briefly, paraffin-embedded sections were incubated with proteinase K (20 µg mL^-1^) for 20 minutes, followed by PBS washes. The sections were incubated with the TUNEL detection solution at 37°C for one hour. Fluorescent images were obtained using a confocal laser scanning microscope (Olympus).

**Enzyme-Linked Immunosorbent Assay (ELISA)**

Chondrocyte-produced collagen Type II and glycosaminoglycans were measured using Mouse ELISA Kits (Fantaibio, Shanghai, China). Briefly, cell supernatants were collected, centrifuged, and purified. An HRP-conjugated reagent was then added and incubated for 60 minutes at 37°C. Following washing, Chromogen Solutions A and B were applied and incubated for 15 minutes at 37°C. The readouts were obtained using a microplate reader (Thermo Fisher Scientific).

**Femoral Head Explant Culture**

Mouse femoral heads were harvested and cultured in DMEM/F12 medium with 10% FBS at 37°C. The femoral heads were pre-incubated with TEPP-46 for seven days, followed by stimulation with 10 ng ml^-1^ IL-1β for another seven days.

**Alcian Blue Staining**

To assess the presence of sulfate proteoglycan, primary chondrocytes were cultured for two days and stained with Alcian blue (Servicebio). In brief, the cells were fixed with 4% paraformaldehyde, followed by a 30-minute incubation with 1% Alcian blue prepared in 1 N HCl.

**Micro-CT Analysis**

Subchondral bone remodeling in the mouse knee joint was assessed using micro-CT. The isolated joint tissues were fixed in 4% paraformaldehyde for 24 hours. The samples were then scanned with a high-resolution micro-CT system (Bruker microCT, Kontich, Belgium) at 55 kV and 145 μA with an isotropic resolution of 5 μm³. The bone volume (BV) and subchondral bone plate (SBP) thickness of the metaphyseal trabecular bone in the proximal tibia were analyzed using three-dimensional model visualization software and data analysis software.

**Western Blotting**

Chondrocytes were lysed in RIPA buffer (Beyotime) containing phosphatase and protease inhibitors (Beyotime). The total cellular proteins were separated by SDS-polyacrylamide gel electrophoresis (SDS-PAGE) and transferred to polyvinylidene fluoride (PVDF) membranes (0.2 μm, Millipore, Billerica, USA). The membranes were blocked with 5% nonfat milk (Beyotime) for one hour at room temperature, followed by overnight incubation at 4°C with primary antibodies (antibody information listed in Supplementary Table 5). After washing, the membranes were incubated with HRP-conjugated secondary antibodies for one hour. Protein bands were visualized using a Tanon Imager 4600 system (Tanon, Shanghai, China).

**JC-1 Mitochondrial Membrane Potential Assay**

Mitochondrial membrane potential (∆Ψm) was assessed using the JC-1 probe (Beyotime). Chondrocytes were seeded in a confocal imaging dish and incubated with JC-1 solution at 37℃ for 20 minutes, following the manufacturer's protocol. Chondrocytes were analyzed using a confocal laser scanning microscope (Olympus). The red-to-green fluorescence ratio, representing J-aggregates (red) versus J-monomers (green), was used to evaluate mitochondrial membrane polarization.

**MitoSOX Staining for Mitochondria-Derived Superoxide Detection**

Mitochondria-derived superoxide levels were detected using the MitoSOX Red mitochondrial superoxide indicator (#M36008, Invitrogen). Cells were incubated with 500 nM MitoSOX Red working solution in Hanks Balanced Salt Solution (HBSS) at 37℃ for 30 minutes. Cells were then examined by confocal microscopy (Olympus).

**Transmission Electron Microscopy (TEM) for Mitochondria**

Mitochondrial ultrastructure was analyzed using transmission electron microscopy. Cells were collected and fixed in 2.5% glutaraldehyde at 4°C for two hours. Following fixation, the samples underwent post-fixation in 1% osmium tetroxide for one hour, then dehydrated through a graded ethanol series and embedded in epoxy resin. Ultrathin sections (70 nm-thick) were cut with an ultramicrotome (Leica) and mounted on copper grids. The sections were subsequently stained with uranyl acetate and lead citrate, and examined using a transmission electron microscope (JEM-1400, JEOL, Japan). Mitochondrial features, such as cristae structure and membrane integrity, were assessed at magnifications ranging from 10,000× to 30,000×.

**Bulk and Single-cell Sequencing Data Processing**

For bulk sequencing, transcriptome data of OA models were acquired from the Gene Expression Omnibus (GEO) database. Transcriptomes of murine OA cartilage (GSE53857) and knee joints (GSE26475), rat OA meniscus (GSE241794), IL-1β-treated cartilage explants (GSE100083 and GSE110754), and IL-1β-treated chondrocytes (GSE75181, GSE6119, and GSE104793) were analyzed.

Raw single-cell RNA sequencing (scRNA-seq) data (GSE104782 and GSE220243) were obtained from the GEO database and processed using the Seurat package (v.5.1.0) in R (v.4.4.1). Cells with a minimum expression of 200 genes and genes expressed in at least three cells were included in the analysis. Normalization and batch effect correction were conducted using Seurat's SCTransform function. Principal component analysis (PCA) was conducted on variable genes based on the RunPCA function. The top 30 principal components were selected for uniform manifold approximation and projection (UMAP) and Louvain clustering through the FindNeighbors, FindClusters, and RunUMAP packages in Seurat. Harmony algorithm (v1.2.0) was applied to integrate cells from both OA and control groups. Clusters were identified by differentially expressed genes (DEGs) and manually annotated using established cell type markers. Gene expression, including *PKM* levels across clusters, was visualized using dot plots. The gene ontology (GO) enrichment analysis was performed using the enrichGO function from the clusterProfiler package in R.

**GSEA**

Chondrocytes transfected with siNC or siPkm2 underwent RNA-seq analysis, as previously reported[5]. DEGs were identified based on a threshold of a fold change greater than 2 and an adjusted p-value less than 0.05. Gene set enrichment analysis (GSEA) was performed using the OmicStudio online tools (https://www.omicstudio.cn/tool). Data visualization, including heatmaps and other plots, was conducted using the pheatmap and ggplot2 packages in R/Bioconductor.

**Reference**

1. Glasson SS, Blanchet TJ, Morris EA. The surgical destabilization of the medial meniscus (DMM) model of osteoarthritis in the 129/SvEv mouse. Osteoarthritis Cartilage. 2007; 15: 1061–9.

2. Mankin HJ, Dorfman H, Lippiello L, Zarins A. Biochemical and metabolic abnormalities in articular cartilage from osteo-arthritic human hips. II. Correlation of morphology with biochemical and metabolic data. The Journal of bone and joint surgery American volume. 1971; 53: 523–37.

3. Glasson SS, Chambers MG, Van Den Berg WB, Little CB. The OARSI histopathology initiative - recommendations for histological assessments of osteoarthritis in the mouse. Osteoarthritis Cartilage. 2010; 18 Suppl 3: S17–23.

4. Krenn V, Morawietz L, Burmester GR, Kinne RW, Mueller-Ladner U, Muller B, et al. Synovitis score: discrimination between chronic low-grade and high-grade synovitis. Histopathology. 2006; 49: 358–64.

5. Liu B, Wang C, Weng Z, Yang Y, Zhao H, Zhang Y, et al. Glycolytic enzyme PKM2 regulates cell senescence but not inflammation in the process of osteoarthritis. Acta Biochim Biophys Sin (Shanghai). 2023; 55: 1425–33.

***PKM* expression in human osteoarthritis (OA) cartilage**


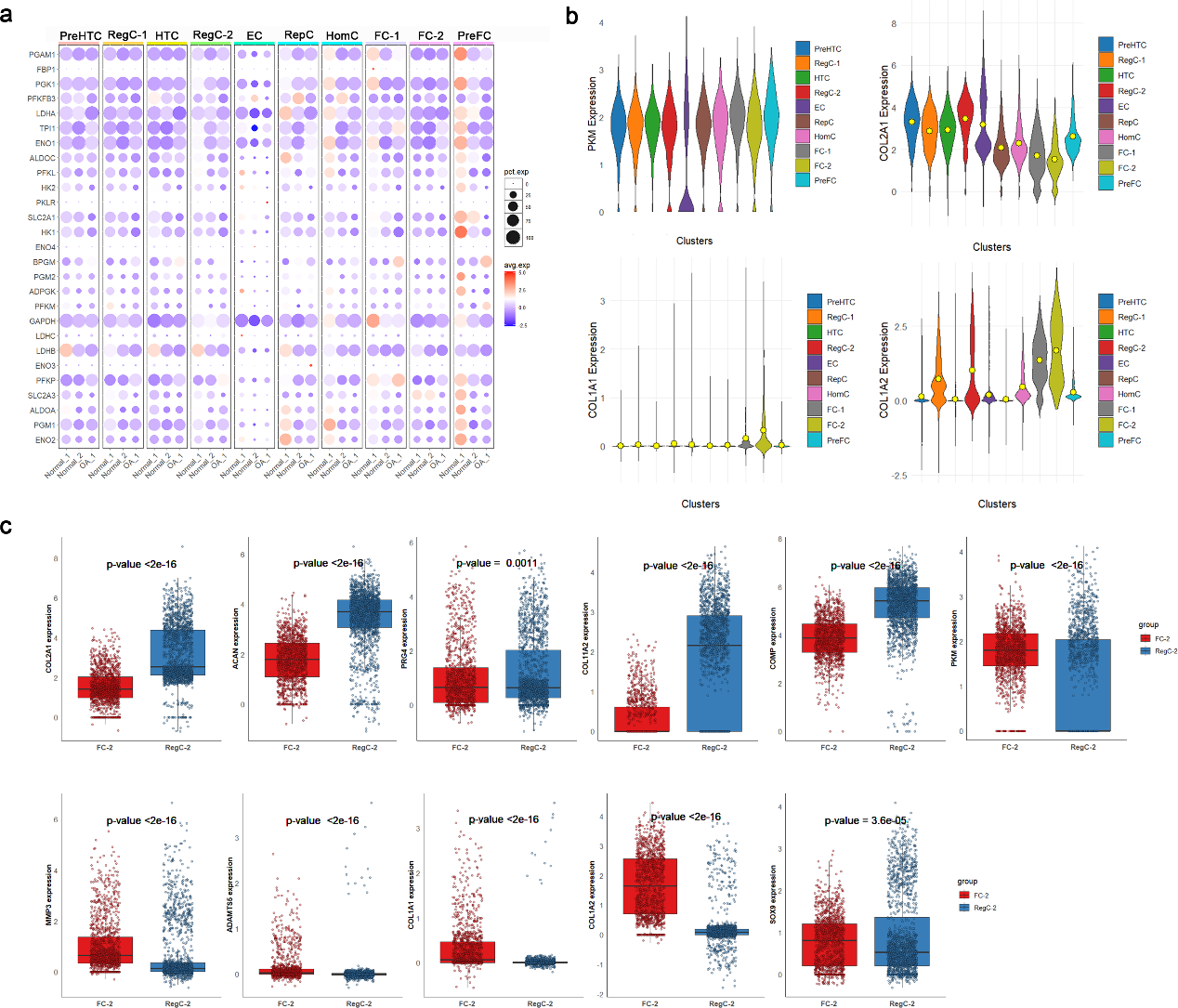
**Fig. S1.** ***PKM* is upregulated in human OA cartilage** **(GSE220243).** **(a)** Dot plots showing transcriptional levels of glycolytic enzymes across subclusters in each sample. **(b)** Violin plots displaying *PKM*, *COL2A1*, *COL1A1* and *COL1A2* expression across subclusters. **(c)** Boxplots comparing *COL2A1*, *ACAN*, *PRG4*, *COL11A2*, *COMP*, *PKM*, *MMP3*, *ADAMTS5*, *COL1A1*, *COL1A2,* and *SOX9* expression between FC-2 and RegC-2 subclusters.

**Upregulation of PKM2, p16^INK4a^, and p21 in osteoarthritic cartilage.**

The protein levels of PKM2, p16^INK4a^, and p21 are elevated in osteoarthritic cartilage.

In cartilage tissue obtained from clinical samples, the protein levels of COL2A1 and ACAN were reduced, while the level of MMP13 was increased in osteoarthritic cartilage (Supplementary Figure 2a, b). PKM2 is significantly upregulated in all layers of osteoarthritic cartilage, including the superficial, middle, and deep layers. (Supplementary Figure 2c). Eight weeks after DMM surgery, the protein levels of COL2A1 and ACAN were reduced, while the protein levels of p16^INK4a^ and p21, two well-known markers of cellular senescence, were elevated (Supplementary Figure 2d, e). The protein levels of p16^INK4a^ and p21 were increased in the cartilage of aged mice (Supplementary Figure 2f, g). These findings suggest that disrupted ECM metabolism and chondrocyte senescence are observed in osteoarthritic cartilage in both human and mouse samples.


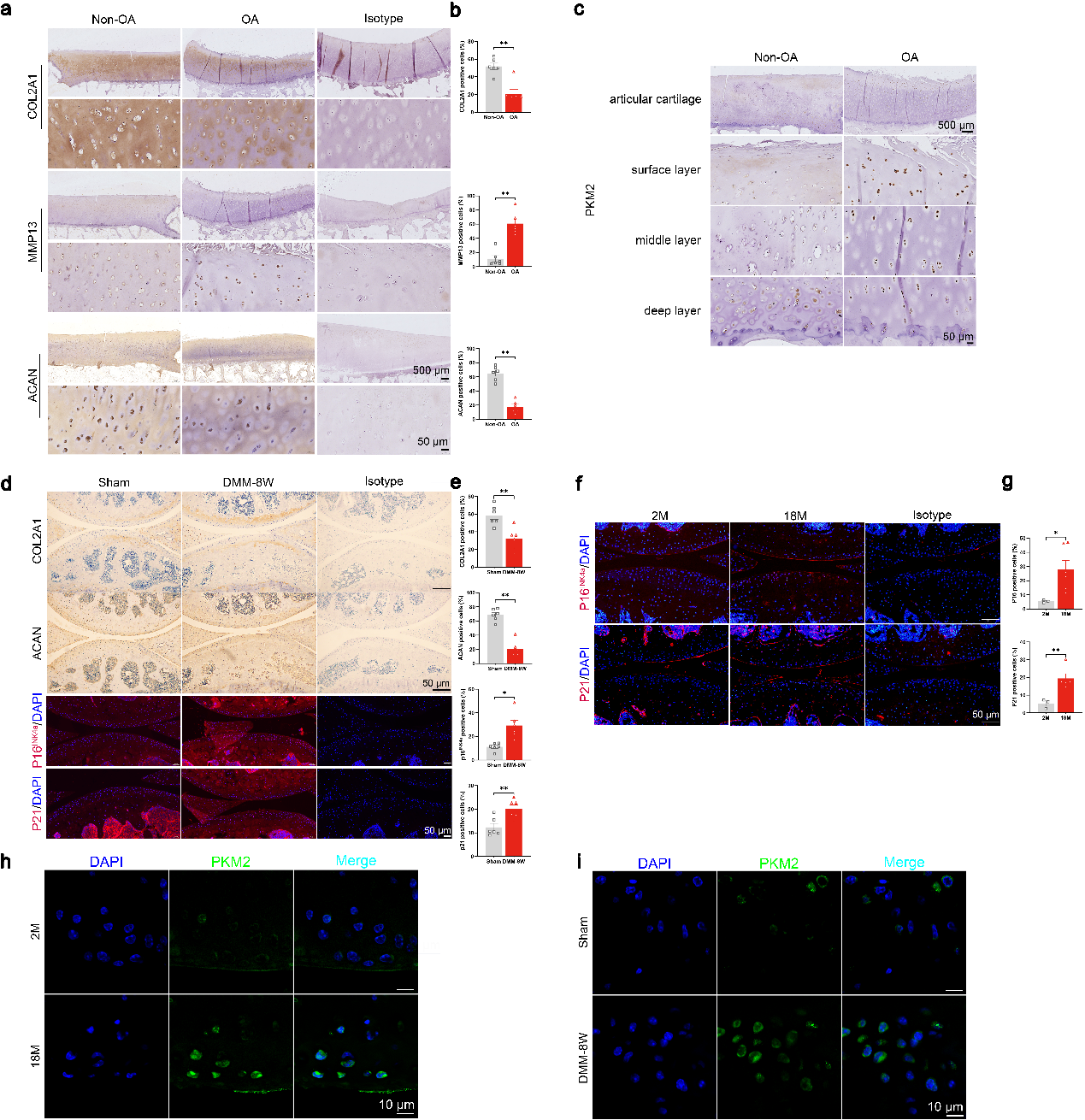
 **Fig. S2.** **Upregulation of PKM2, p16^INK4a^, and p21 in osteoarthritic cartilage.**

**(a)** Representative IHC images and (**b)** quantification of COL2A1, ACAN, and MMP13 in human non-OA and OA cartilage. (**c)** Representative IHC staining of PKM2 in human non-OA and OA cartilage. **(d)** Representative IHC staining and **(e)** quantification of COL2A1, ACAN, p16^INK4a^, and p21 in mouse cartilage eight weeks after DMM surgery. (**f)** Representative IF images and **(g)** quantification of p16^INK4a^ and p21 in cartilage from 2-month-old and 18-month-old mice. **(h)** Representative IF staining of PKM2 in cartilage from 2-month-old and 18-month-old mice. (**i)** Representative IF staining of PKM2 in mouse cartilage eight weeks after DMM surgery. Data are presented as means ± s.e.m., n =6, unpaired Student's t-test. *P < 0.05, **P < 0.01.

**Upregulation of PKM2 in osteoarthritic meniscus.**

PKM2 levels were detected in articular cartilage, the surface layer of the medial meniscus, and bone marrow, but not in the synovium (Supplementary Figure 3a). PKM2 expression was upregulated in the meniscus tissue of mice with surgery-induced and aging-induced osteoarthritis (OA) (Supplementary Figure 3b). Additionally, analysis of online data (GSE241794) revealed increased PKM expression in the meniscus tissues of surgery-induced OA rats (Supplementary Figure 3c). These findings suggest that PKM2 expression is elevated in both cartilage and meniscus tissues during the progression of OA.

**
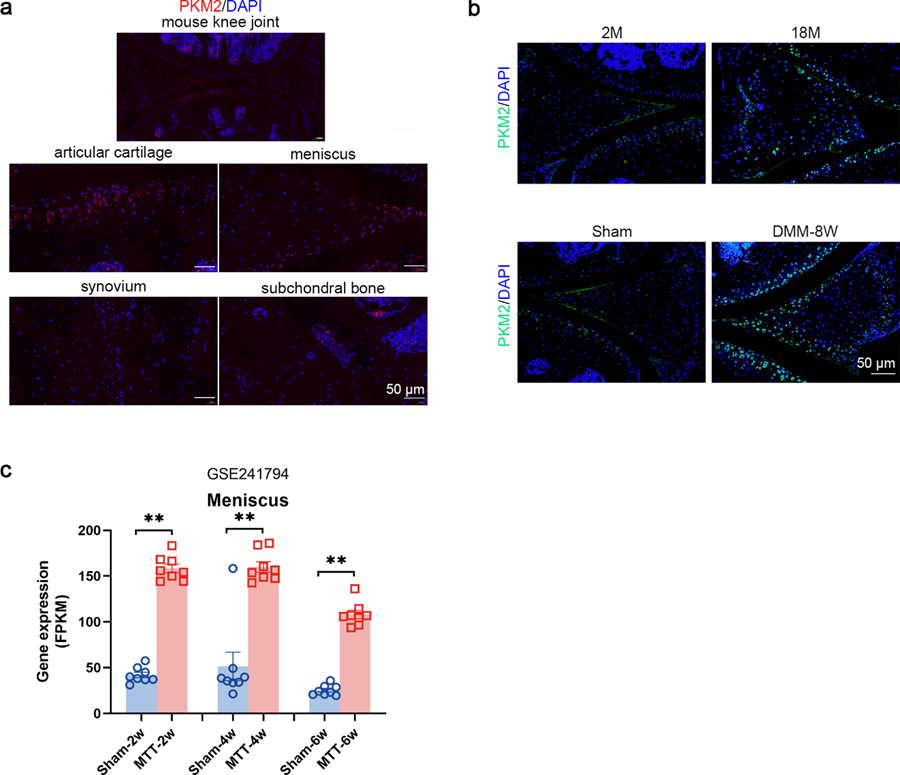
Fig. S3.** **Upregulation of PKM2 in osteoarthritic meniscus. (a)** Representative IF staining of PKM2 in the knee joints (cartilage, meniscus, synovium, and subchondral bone) of 2-month-old mouse. **(b)** Representative IF staining of PKM2 in the meniscus of mice with DMM surgery-induced or aging-associated OA. **(c)** Quantification of *PKM* expression in rat meniscus tissue at two, four, and six weeks post-medial meniscus transection (MMT) surgery, using a publicly available dataset (GSE241794), shown as Fragments Per Kilobase of exon per Million mapped reads (FPKM). Data are presented as means ± s.e.m., n=8, paired Student's t-test. **P < 0.01.

**Identification of primary chondrocytes and** **validation of siRNA knockdown and ECM gene expression.**

Primary mouse chondrocytes were isolated and validated based on morphology, Alcian blue staining, and the protein expression of ACAN and COL2A1 (Supplementary Figure 4a). Three designed siPkm2 were transfected into cultured chondrocytes, resulting in a significant reduction of PKM2 mRNA and protein expression (Supplementary Figure 4b-d). Additionally, mRNA expression levels of COL2A1, ACAN, cartilage oligomeric matrix protein (COMP), collagen type IX alpha 1 chain (COL9A1), collagen type XI alpha 2 chain (COL11A2), MMP3, MMP13, and ADAMTS5 were measured in cultured chondrocytes in the absence (Supplementary Figure 4e) or presence (Supplementary Figure 4f) of IL-1β. Taken together, the third siPkm2 was selected in the present study.


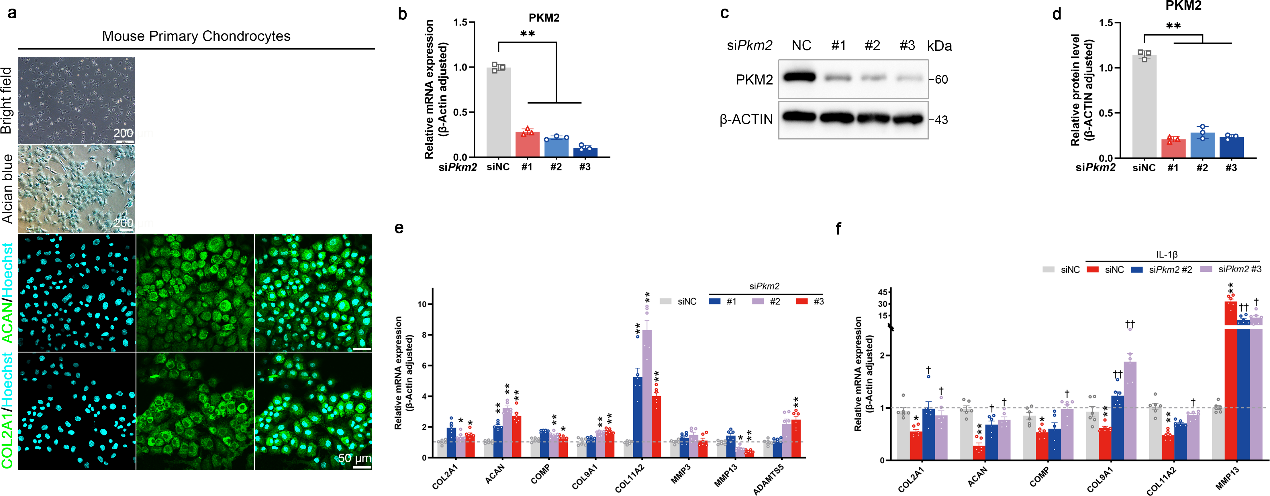
 **Fig. S4.** **Identification of primary chondrocytes and validation of siRNA knockdown and ECM gene expression. (a)** Primary mouse chondrocytes were identified based on morphology, Alcian blue staining, and IF staining of ACAN and COL2A1. ACAN and COL2A1 signals are labeled in cyan green, and nuclei are counterstained using Hoechst. **(b)** Relative PKM2 mRNA expression in chondrocytes transfected with siNC or siPkm2. Data are presented as means ± s.e.m., n=3, one-way ANOVA with Dunnett's comparisons. **(c)** Representative immunoblots and **(d)** densitometric quantification of PKM2 in chondrocytes transfected with siNC or siPkm2. Data are presented as means ± s.e.m., n=3, one-way ANOVA with Dunnett's comparisons. **(e)** Relative mRNA expression levels of COL2A1, ACAN, COMP, COL9A1, COL11A2, MMP3, MMP13, and ADAMTS5 in chondrocytes transfected with siNC or siPkm2 under basal conditions. Data are presented as means ± s.e.m., n = 6, one-way ANOVA with Dunnett's comparisons. **(f)** Relative mRNA expression levels of COL2A1, ACAN, COMP, COL9A1, COL11A2, and MMP13 in IL-1β-stimulated chondrocytes transfected with siNC or siPkm2. Data are presented as means ± s.e.m., n = 6, one-way ANOVA with Tukey’s multiple comparisons. *P < 0.05, **P < 0.01 compared to siNC; †P < 0.05, ††P < 0.01 compared to siNC+IL-1β.

**Generation and validation of cartilage-specific Pkm2^icKO^ mice.**

Two-week-old mice were used to confirm the gene mutation, and they were euthanized after tamoxifen administration (Supplementary Figure 5h). There were no significant differences in body size between Pkm2^fl/fl^ and Pkm2^icKO^ mice at the developmental stage (Supplementary Figure 5i). Deletion of PKM2 resulted in a slight reduction in the thickness of both articular cartilage and growth plates (Supplementary Figure 5j). Further investigation revealed that chondrocyte proliferation and apoptosis were unaffected by PKM2 depletion at this developmental stage (Supplementary Figure 5j).


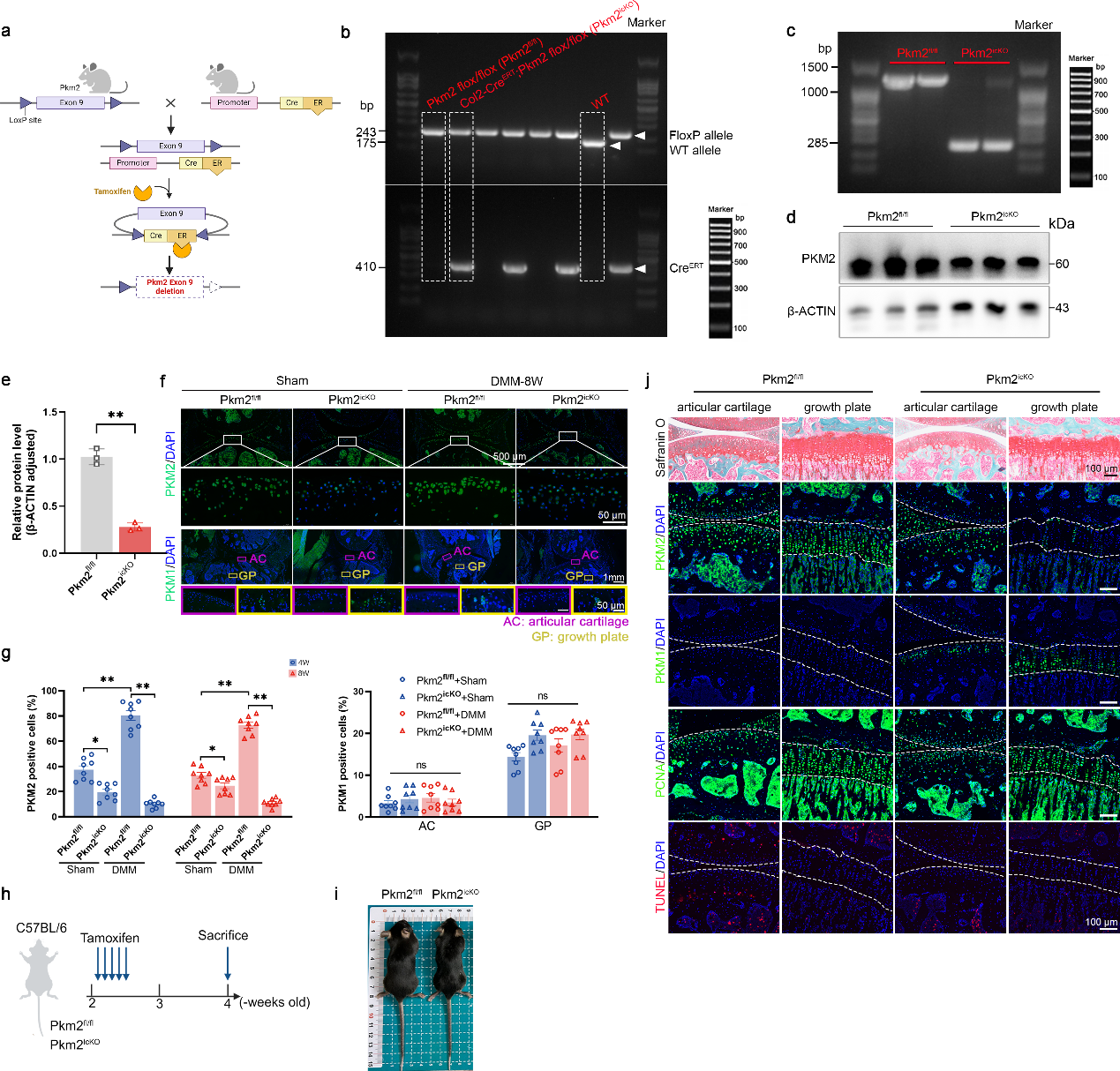
 **Fig. S5.** **Generation and validation of cartilage-specific Pkm2^icKO^ mice.**

**(a)** Schematic illustration of the strategy used to generate Pkm2^icKO^ mice. Exon 10, which is specific to the PKM2 isoform, was targeted to achieve conditional deletion of PKM2 in chondrocytes. **(b****)** PCR genotyping of tail samples from Pkm2^fl/fl^ and Pkm2^icKO^ mice. A 243-bp band was detected in homozygous Pkm2^fl/fl^ and Pkm2^icKO^ mice, whereas a 175-bp band was detected in wild-type (WT) mice. (**c)** PCR genotyping of cartilage samples from Pkm2^fl/fl^ and Pkm2^icKO^ mice. Chondrocyte-specific PKM2 deletion was further verified using an additional primer set, which yielded a 285-bp band in cartilage tissues with Cre activity in Pkm2^icKO^ mice. **(d)** Representative immunoblots and **(e)** densitometric quantification of PKM2 levels in the cartilage tissue of adult Pkm2^fl/fl^ and Pkm2^icKO^ mice. Data are presented as means ± s.e.m., n = 3, unpaired Student’s t-test. **(f)** Representative IF staining and **(g)** quantification of PKM2 and PKM1 in the articular cartilage and growth plates of adult Pkm2^fl/fl^ and Pkm2^icKO^ mice. Data are presented as means ± s.e.m., n = 8, one-way ANOVA with Tukey’s multiple comparisons. **(h)** Schematic diagram of cartilage-specific PKM2 deletion during development. Tamoxifen was administered for five consecutive days to two-week-old mice, and cartilage tissues were collected at four weeks of age for histological analyses. **(i)** General appearance of Pkm2^fl/fl^ and Pkm2^icKO^ mice. **(j)** Representative SO&FG staining, IF staining of PKM2, PKM1, PCNA, and TUNEL staining in the tibial articular cartilage and growth plates of Pkm2^fl/fl^ and Pkm2^icKO^ mice. **P < 0.01. ns, not significant.

**PKM2 deletion promotes chondrocyte proliferation and inhibits apoptosis**


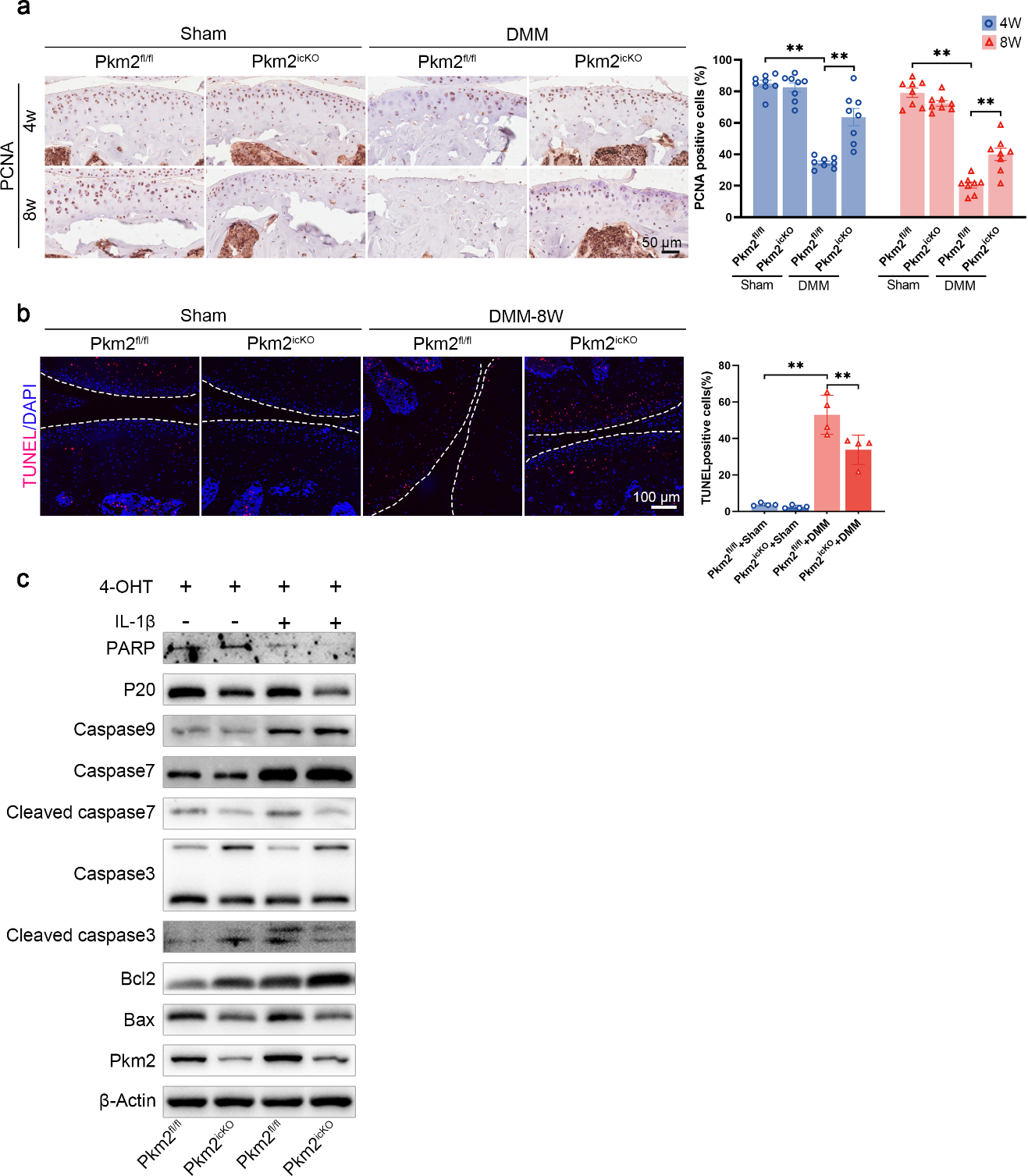


**Fig. S6.** **PKM2 deletion promotes chondrocyte proliferation and inhibits apoptosis.** **(a)** Representative IHC staining and quantification of PCNA in the cartilage of Pkm2^fl/fl^ and Pkm2^icKO^ mice, four and eight weeks after surgery. Data are presented as means ± s.e.m., n = 6, one-way ANOVA with Tukey's multiple comparisons. **(b)** Representative TUNEL staining and quantification of TUNEL-positive chondrocytes in the cartilage of Pkm2^fl/fl^ and Pkm2^icKO^ mice, four and eight weeks after surgery. Data are presented as means ± s.e.m., n = 6, one-way ANOVA with Tukey's multiple comparisons. **P < 0.01.

**PKM2 deficiency reduces p16^INK4a^ and p21 levels in osteoarthritic cartilage**

**
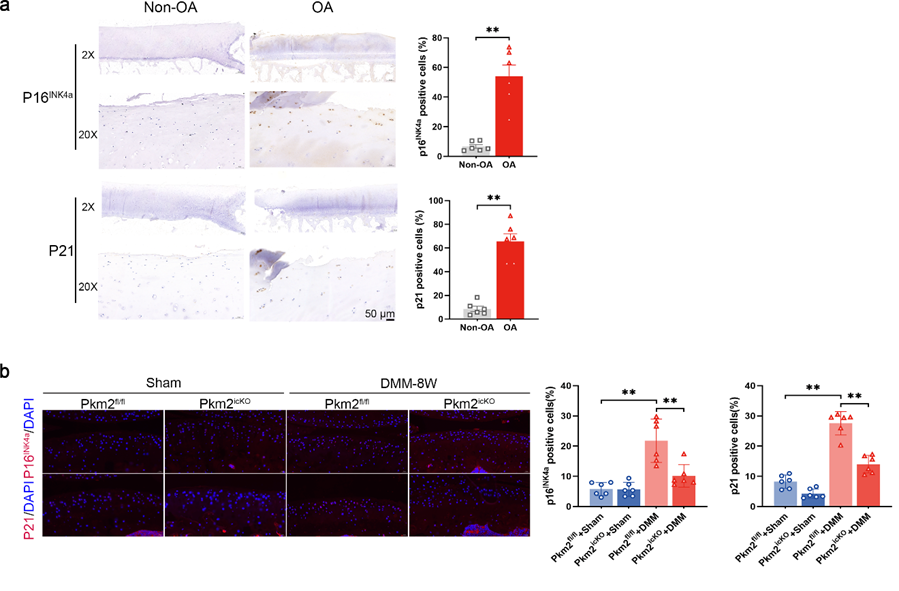
Fig. S7.** **PKM2 deficiency reduces p16^INK4a^ and p21 levels in osteoarthritic cartilage.** **(a)** Representative IHC staining and quantification of p16^INK4a^ and p21 in human non-OA and OA cartilage. Data are presented as means ± s.e.m., n = 6, unpaired Student's t-test. (**b)** Representative IF staining and quantification of p16^INK4a^ and p21 in the cartilage of Pkm2^fl/fl^ and Pkm2^icKO^ mice eight weeks after DMM surgery. Data are presented as means ± s.e.m., n = 6, one-way ANOVA with Tukey's multiple comparisons. **P < 0.01.

**PKM2 deletion alleviates surgery-related subchondral bone sclerosis**


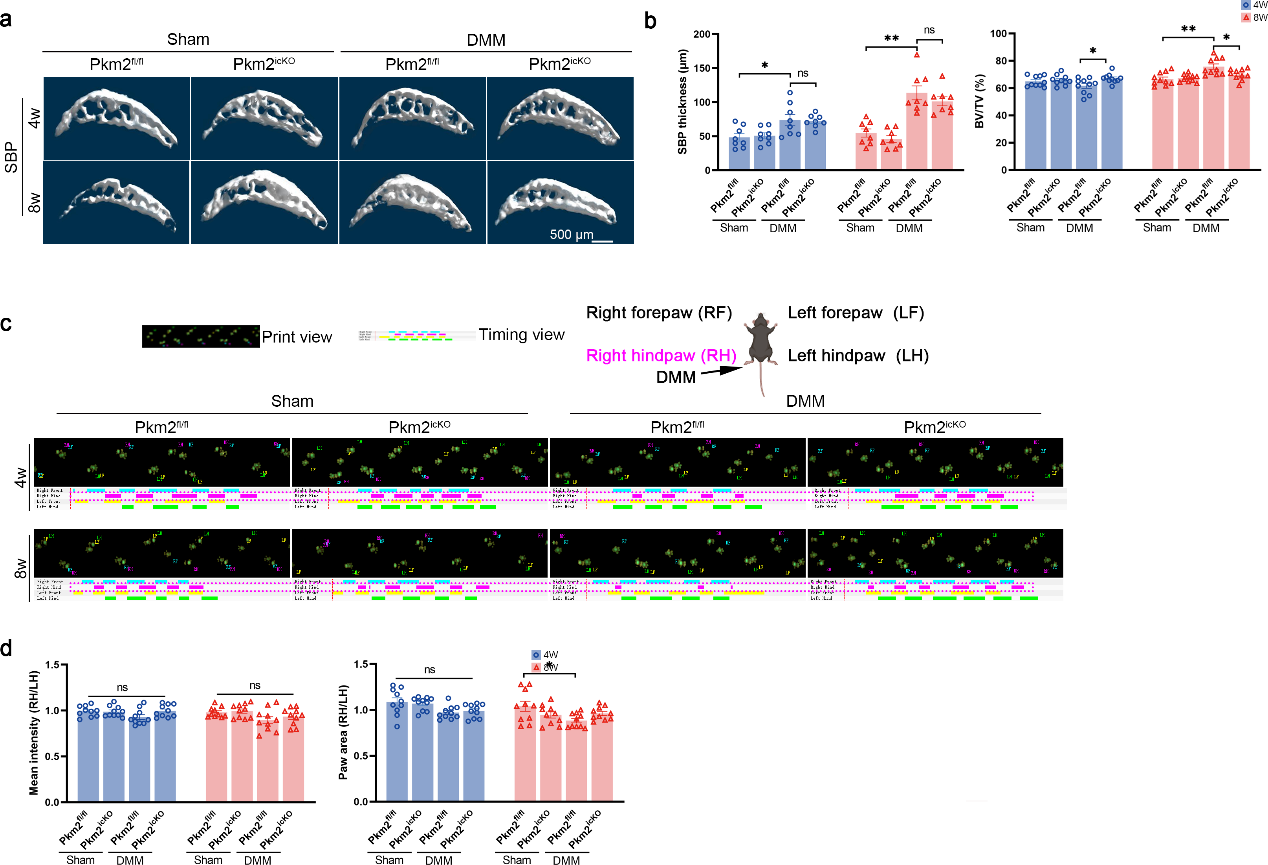
 **Fig. S8.** **PKM2 deletion alleviates DMM surgery-** **induced subchondral bone sclerosis. (a)** Representative coronally reconstructed micro-CT images of medial tibial subchondral bone in Pkm2^fl/fl^ and Pkm2^icKO^ mice at four weeks and eight weeks post-DMM surgery. (**b)** Quantification of subchondral bone plate thickness (SBP) and bone volume fraction (Bone Volume/Total Volume, BV/TV). Data are presented as means ± s.e.m., n = 8, one-way ANOVA with Tukey’s multiple comparisons. (**c)** CatWalk gait analysis showing representative paw-print traces in adult Pkm2^fl/fl^ and Pkm2^icKO^ mice four and eight weeks after DMM surgery. **(d)** Quantification of hind-limb movement parameters (mean intensity and paw area), presented as the RH/LH ratio. Data are presented as means ± s.e.m., n = 8, one-way ANOVA with Tukey’s multiple comparisons. *P < 0.05, **P < 0.01. ns, not significant.


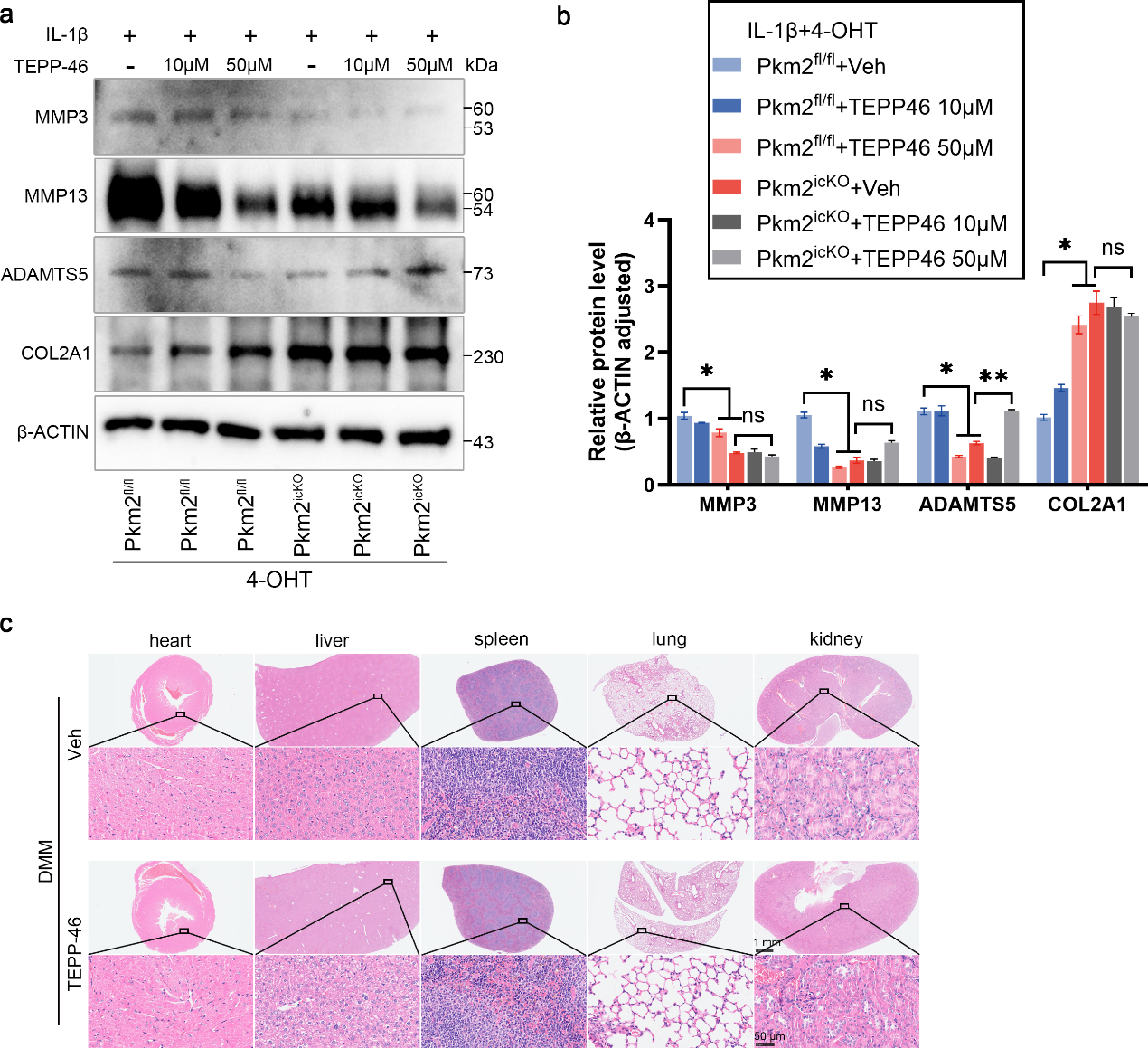
 **TEPP-46 maintains ECM homeostasis via PKM2 and exhibits no systemic toxicity.**

**Fig. S9.** **TEPP-46 maintains ECM homeostasis via PKM2 and exhibits no systemic toxicity. (a)** Representative immunoblots and **(b)** densitometric quantification of MMP3, MMP13, ADAMTS5, and COL2A1 in chondrocytes isolated from Pkm2^fl/fl^ and Pkm2^icKO^ mice, treated with TEPP-46 in the presence of IL-1β and 4-OHT. Data are presented as means ± s.e.m., n = 3, one-way ANOVA with Tukey’s multiple comparisons. **(c)** Representative hematoxylin and eosin (H&E) staining of the heart, liver, spleen, lung, and kidneys from mice injected with vehicle or TEPP-46 (50 μM) eight weeks after DMM surgery.

**TEPP-46 alleviates DMM surgery-induced subchondral bone sclerosis.**


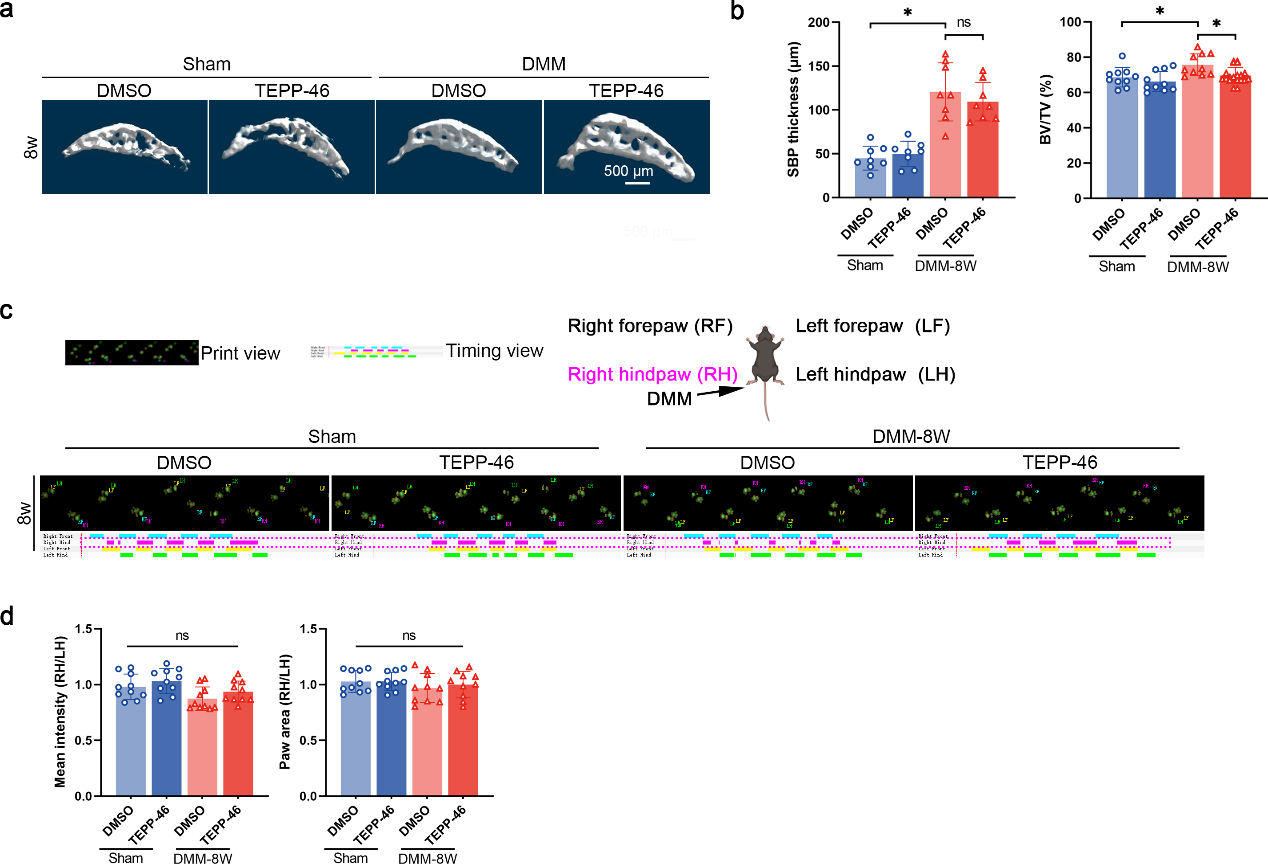
 **Fig. S10. TEPP-46 alleviate DMM surgery-** **induced subchondral bone sclerosis. (a)** Representative coronally reconstructed micro-CT images of medial tibial subchondral bone in mice treated with TEPP-46 eight weeks after DMM surgery. (**b)** Quantification of SBP and bone volume fraction (BV/TV) in mice treated with TEPP-46 eight weeks post-DMM surgery. Data are presented as means ± s.e.m., n = 8, one-way ANOVA with Tukey’s multiple comparisons. (**c)** Representative CatWalk gait traces from mice treated with TEPP-46 eight weeks after DMM surgery. **(d)** Quantification of hind-limb gait parameters (mean intensity and paw area) in mice treated with TEPP-46, presented as the RH/LH ratio. Data are presented as means ± s.e.m., n = 8, one-way ANOVA with Tukey’s multiple comparisons. *P < 0.05, **P < 0.01. ns, not significant.


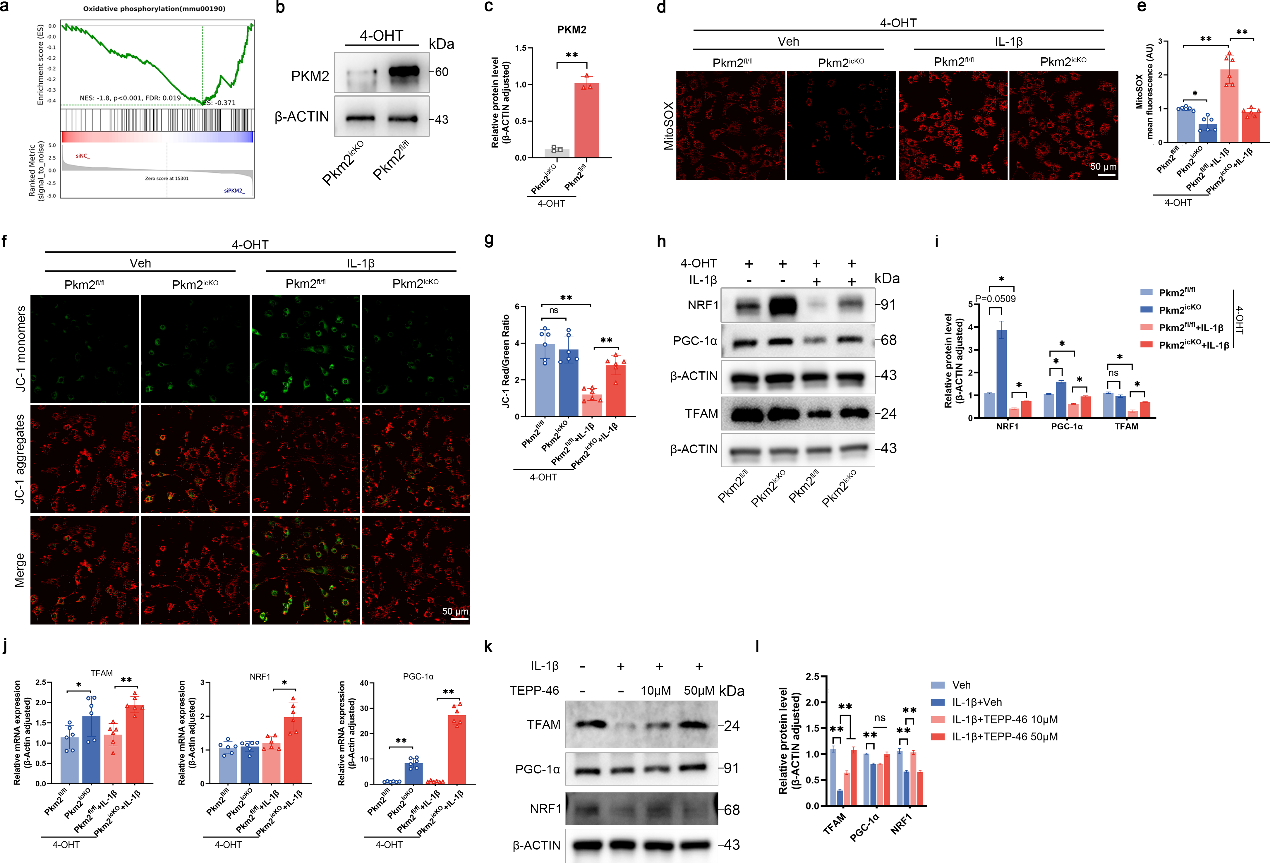
**PKM2 deletion preserves mitochondrial integrity and bioenergetic function in chondrocytes.**

**Fig. S11.** **PKM2 deletion preserves mitochondrial integrity and bioenergetic function in chondrocytes.** (**a)** GSEA highlighting the oxidative phosphorylation pathway in chondrocytes transfected with siPkm2. **(b)** Representative immunoblots and **(c)** densitometric quantification of PKM2 in chondrocytes isolated from adult Pkm2^fl/fl^ and Pkm2^icKO^ mice and incubated with 4-OHT. Data are presented as means ± s.e.m., n = 3, paired Student’s t-test. **(d)** Representative images of mitochondrial ROS staining and **(e)** quantification in IL-1β-stimulated chondrocytes isolated from Pkm2^fl/fl^ and Pkm2^icKO^ mice in the presence of 4-OHT. Data are presented as means ± s.e.m., n=6, one-way ANOVA with Tukey's comparisons. **(f)** Representative JC-1 staining of mitochondrial membrane potential in chondrocytes IL-1β-stimulated isolated from Pkm2^fl/fl^ and Pkm2^icKO^ in the presence of 4-OHT (J-monomers in green; J-aggregates in red). **(g)** Quantification of mitochondrial membrane potential, presented as the ratio of J-monomer to J-aggregate fluorescence intensity. Data are presented as means ± s.e.m., n=6, one-way ANOVA with Tukey's comparisons. **(h)** Representative immunoblots and **(i)** densitometric quantification of TFAM, NRF1, and PGC-1α in IL-1β-stimulated chondrocytes isolated from Pkm2^fl/fl^ and Pkm2^icKO^ mice in the presence of 4-OHT. Data are presented as means ± s.e.m., n=6, one-way ANOVA with Tukey's comparisons. **(j)** Relative mRNA expression of TFAM, NRF1, and PGC-1α in IL-1β-stimulated chondrocytes isolated from Pkm2^fl/fl^ and Pkm2^icKO^ mice in the presence of 4-OHT. Data are presented as means ± s.e.m., n=6, one-way ANOVA with Tukey's comparisons. **(k)** Representative immunoblots and **(l)** densitometric quantification of TFAM, PGC-1α, and NRF1 in IL-1β-stimulated chondrocytes incubated with vehicle or TEPP-46 (10, 50 μM). Data are presented as means ± s.e.m., n=6, one-way ANOVA with Tukey's comparisons. *P < 0.05, **P < 0.01, ***P < 0.001. ns, not significant.


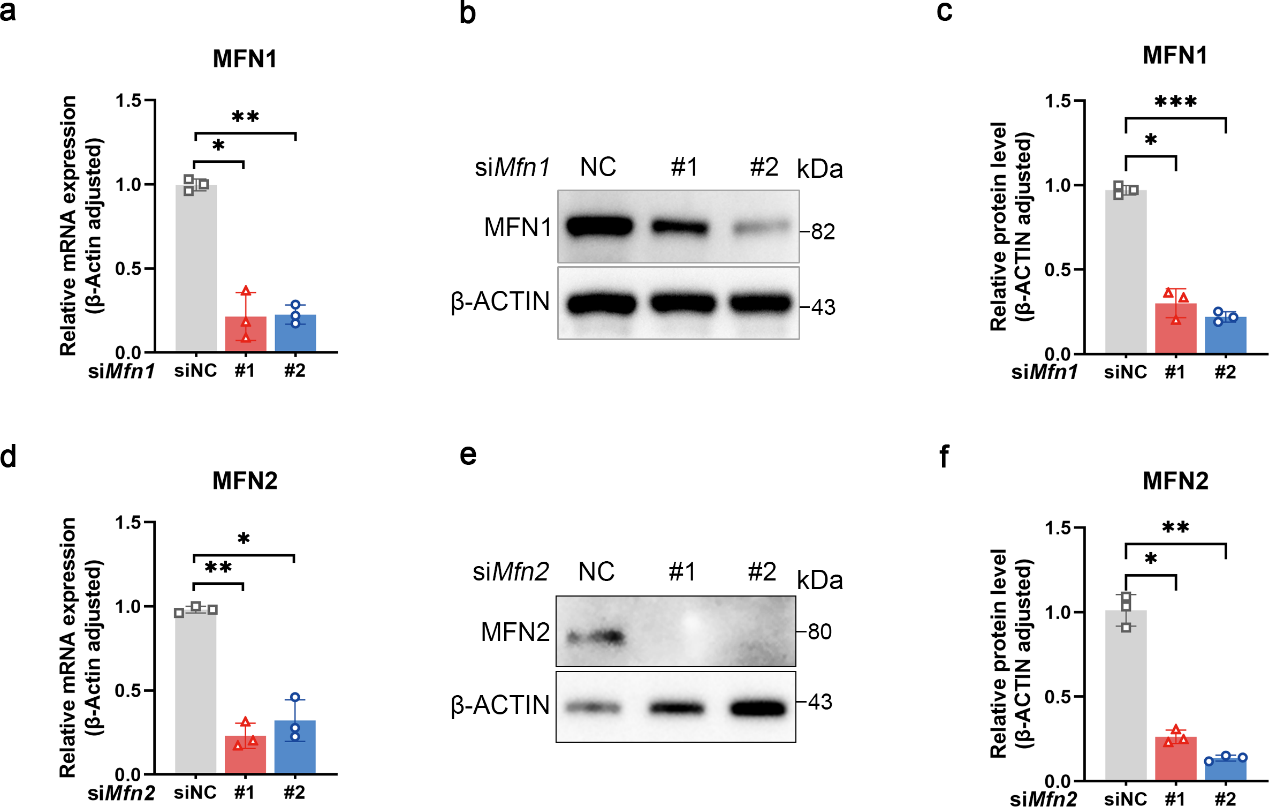
**Validation of siMFN1 and siMFN2 knockdown.**

**Fig. S12.** **Validation of siMFN1 and siMFN2 knockdown.**

**(a)** Relative MFN1 mRNA expression in chondrocytes transfected with siNC or si*Mfn1*. Data are presented as means ± s.e.m., n=3, one-way ANOVA with Dunnett's comparisons. **(b)** Representative immunoblots and **(c)** densitometric quantification of MFN1 in chondrocytes transfected with siNC or si*Mfn1*. Data are presented as means ± s.e.m., n=3, one-way ANOVA with Dunnett's comparisons. **(d)** Relative MFN2 mRNA expression in chondrocytes transfected with siNC or si*Mfn2*. Data are presented as means ± s.e.m., n=3, one-way ANOVA with Dunnett's comparisons. **(e)** Representative immunoblots and **(f)** densitometric quantification of MFN2 in chondrocytes transfected with siNC or si*Mfn2*. Data are presented as means ± s.e.m., n=3, one-way ANOVA with Dunnett's comparisons. *P < 0.05, **P < 0.01, ***P < 0.001.

**MFN1 expression was decreased in osteoarthritic cartilage.**
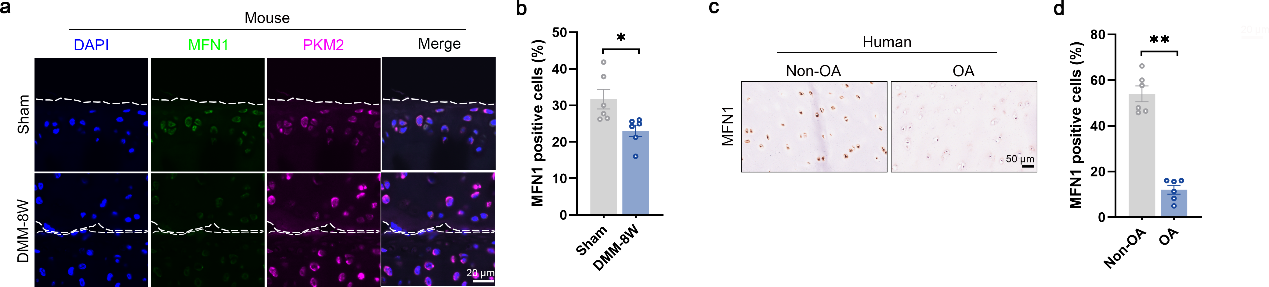


**Fig. S13.** **MFN1 expression was decreased in osteoarthritic cartilage.**

**(a)** Representative IF staining and **(b)** quantification of MFN1 and PKM2 in the cartilage of 2-, 10- and 18-month-old mice. Data are presented as means ± s.e.m., n = 6, one-way ANOVA with Dunnett's comparisons. (**c)** Representative IF staining and (**d)** quantification of MFN1 and PKM2 in the cartilage of mice eight weeks after DMM surgery. Data are presented as means ± s.e.m., n = 6, unpaired Student's t-test. *P < 0.05, **P < 0.01. ns, not significant.

**GO and KEGG enrichment analysis between PKM^high^ and PKM^low^ cells from human OA cartilage single-cell transcriptomic data (GSE104782).**

The online single-cell RNA sequencing dataset (GSE104782) included ten osteoarthritis (OA) cartilage samples obtained from patients undergoing knee arthroplasty. Clusters were identified and annotated based on differentially expressed genes (DEGs) (Supplementary Figure 14a,b). Cells with the highest 10% expression of the *PKM* gene were classified as PKM^high^ cells, while those with the lowest 10% were classified as PKM^low^ cells. The top 50 DEGs between these two clusters are listed (Supplementary Figure 14c). Gene Ontology (GO) enrichment analysis revealed significant modules related to the transmembrane receptor protein serine/threonine kinase signaling pathway, extracellular matrix (ECM) organization, extracellular structure organization, and cartilage development (Supplementary Figure 14 d). Kyoto Encyclopedia of Genes and Genomes (KEGG) enrichment analysis revealed modules related to ECM-receptor interaction.


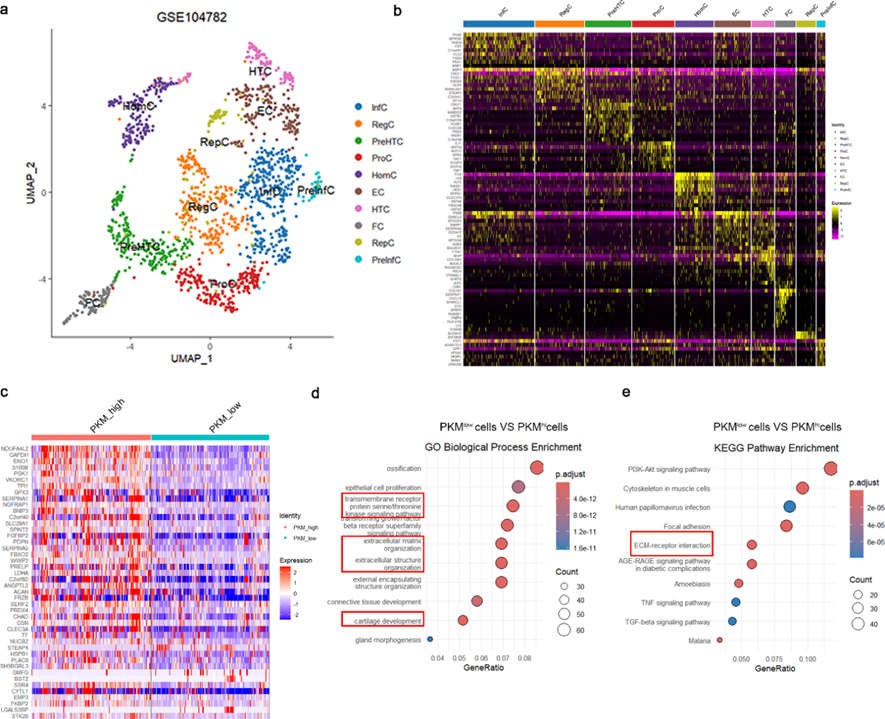
**Fig. S14.** **GO and KEGG enrichment analysis between PKM^high^ and PKM^low^ cells from human OA cartilage single-cell transcriptomic data (GSE104782).**

**(a)** UMAP visualization of the single-cell transcriptomes from human OA cartilage (GSE104782). (**b)** Heatmap showing scaled expression of DEGs across subclusters in GSE104782. (**c)** Heatmaps showing the DEGs between the PKM^low^ and PKM^high^ groups. (**d)** GO enrichment analysis highlighting ECM and MAPK signaling pathways. (**e)** KEGG enrichment analysis associated with ECM-related processes.

| Gene | Primer |
| --- | --- |

| PKM2  Flox:243bp,  WT:175bp | Forward primer (F1):5’-CATTATAGGATAACCAGCTTCTCGC-3’  Reverse primer (R1):5’-TAGAAACTCCACAGGAACCATCC-3’ |
| --- | --- |
| Col2-Cre^ERT^  : 410bp | Forward primer (F2):5’-GGCTCTACTTCATCGCATTCCTTG-3’  Reverse primer (R2): 5’-CGCAAACAAGTCTCACAAAGGAG-3’ |
| Col2-Cre^ERT^ del  With Cre activity: one band with 285 bp | Forward primer (F3): 5’-CATTATAGGATAACCAGCTTCTCGC-3’  Reverse primer (R3): 5’-AATCCTTTATCCACCAAGACCCCT-3’ |

**Supplementary Table 1. Primer sequences used for PCR screening in genotyping**

| scramble siNC | Sense: UUCUCCGAACGUGUCACGUTT  Antisense: ACGUGACACGUUCGGAGAATT |
| --- | --- |
| siPkm2 #1 | Sense: CUGGCAUCAUUUGUACCAUTT  Antisense: AUGGUACAAAUGAUGCCAGTT |
| siPkm2 #2 | Sense: CAGAGACCAUCAAGAAUGUTT  Antisense: ACAUUCUUGAUGGUCUCUGTT |
| siPkm2 #3 | Sense: CAUGCUGUCUGGAGAAACATT  Antisense: UGUUUCUCCAGACAGCAUGTT |
| siMfn1 #1 | Sense: GGAUGUAACCACUACUAAACA  Antisense: UUUAGUAGUGGUUACAUCCUU |
| siMfn1 #2 | Sense: GAAGACUAUUACAAUGUUAUA  Antisense: UAACAUUGUAAUAGUCUUCAA |
| siMfn2 #1 | Sense: GCAGCUCAUUAUCAGUUAUTT  Antisense: AUAACUGAUAAUGAGCUGCTT |
| siMfn2 #2 | Sense: CUGCGAAUUAAGCAGAUUATT  Antisense: UAAUCUGCUUAAUUCGCAGTT |
| AAV-shMfn1 | Sense: GCAGAAGGATTTCAAGCAA  Antisense: TTGCTTGAAATCCTTCTGC |

| Gene | Sequence (5′→ 3′) |
| --- | --- |

**Supplementary Table 2. siRNA and shRNA information (Mouse)**

**Supplementary Table 3. Clinical characteristics of patients**

| Patient | Gender | Age (years) | Weight (Kg) | | TKA/Trauma/Tumor | | Other diseases | | ICRS grade* | |
| --- | --- | --- | --- | --- | --- | --- | --- | --- | --- | --- |
| 1 | Female | 68 | 72 | TKA (Right) | | Hypertension | | III | |  |
| 2 | Male | 70 | 80 | TKA (Left) | | Diabetes, Hypertension | | IV | |  |
| 3 | Female | 63 | 58 | TKA (Right) | | / | | II | |  |
| 4 | Male | 66 | 82 | TKA (Left) | | / | | III | |  |
| 5 | Female | 71 | 66 | TKA (Right) | | Diabetes | | II | |  |
| 6 | Male | 59 | 74 | TKA (Left) | | / | | IV | |  |
| 7 | Male | 48 | 78 | Trauma (Right) | | / | | 0 | |  |
| 8 | Female | 52 | 63 | Tumor (Left) | | Hypertension | | 0 | |  |
| 9 | Male | 45 | 70 | Trauma (Left) | | / | | I | |  |
| 10 | Female | 50 | 68 | Tumor (Right) | | / | | 0 | |  |
| 11 | Male | 41 | 76 | Trauma (Right) | | / | | I | |  |
| 12 | Female | 55 | 60 | Tumor (Left) | | Hypertension | | I | |  |

*ICRS: International Cartilage Repair Society

**Supplementary Table 4. Primer sequences used in qRT-PCR (Mouse)**

| Gene | Primer (5′→ 3′) |
| --- | --- |
| COL2A1 | Forward primer: CATCTTGCCGCATCTGTGTG  Reverse primer: TGCCCCTTTGGCCCTAATTT |
| ACAN | Forward primer: GTGGAGCCGTGTTTCCAAG  Reverse primer: AGATGCTGTTGACTCGAACCT |
| MFN2 | Forward primer: CCAACTCCAAGTGTCCGCTC  Reverse primer: GTCCAGCTCCGTGGTAACATC |
| COMP | Forward primer: GGTGCGGTGCATCAATACCA  Reverse primer: GTTGGACTTAGCGAAGGTCAG |
| COL9A1 | Forward primer: AAGCGTCGTGCAAGATTTCCT  Reverse primer: GGGATACAAATGCCTTGTTGGA |
| COL11A2 | Forward primer: CCGATGGTGTTCGGAGATCAA  Reverse primer: GCTGTATAGAGTCAAGAGGGGA |
| MMP3 | Forward primer: ACACCGGATTTGCCAAGACA  Reverse primer: GACTGTTCCAGGCCCATCAA |
| MMP13 | Forward primer: TGTTTGCAGAGCACTACTTGAA  Reverse primer: CAGTCACCTCTAAGCCAAAGAAA |
| ADAMTS5 | Forward primer: GGAGCGAGGCCATTTACAAC  Reverse primer: CGTAGACAAGGTAGCCCACTTT |
| PKM2 | Forward primer: TTGCAGCTATTCGAGGAACTCCG  Reverse primer: CACGATAATGGCCCCACTGC |
| TFAM | Forward primer: AACACCCAGATGCAAAACTTTCA  Reverse primer: GACTTGGAGTTAGCTGCTCTTT |
| PGC-1α | Forward primer: TATGGAGTGACATAGAGTGTGCT  Reverse primer: GTCGCTACACCACTTCAATCC |
| NRF1 | Forward primer: AGCACGGAGTGACCCAAAC  Reverse primer: AGGATGTCCGAGTCATCATAAGA |
| β-Actin | Forward primer: GGCTGTATTCCCCTCCATCG  Reverse primer: CCAGTTGGTAACAATGCCATGT |
| MFN1 | Forward primer: CCTACTGCTCCTTCTAACCCA  Reverse primer: AGGGACGCCAATCCTGTGA |

**Supplementary Table 5. Antibody information**

| Antibody | Brand (catalog no.) | Application (dilution) |
| --- | --- | --- |
| Rabbit anti-PKM2 | Cell Signaling (#4053) | IHC (1:800), IF (1:100)  WB (1:1000) |
| Rabbit anti-PKM1 | Cell Signaling (#7067) | IHC (1:300), IF (1:100) |
| Rabbit anti-MMP3 | Abcam (ab52915) | IHC (1:50), WB (1:1000) |
| Rabbit anti-MMP13 | Proteintech (18165-1-AP) | IHC (1:200), IF (1:100)  WB (1:1000) |
| Rabbit anti-COL2A1 | Proteintech (28459-1-AP) | IHC (1:800), IF (1:200)  WB (1:500) |
| Rabbit anti-ACAN | Proteintech (13880-1-AP) | IHC (1:200), IF (1:100)  WB (1:500) |
| Mouse anti-SOX9 | Proteintech (67439-1-Ig) | IHC (1:400), WB (1:1000) |
| Rabbit anti-DRP1 | Proteintech (12957-1-AP) | WB (1:1000) |
| Rabbit anti-OPA1 | Proteintech (27733-1-AP) | WB (1:1000) |
| Rabbit anti-FIS1 | Proteintech (10956-1-AP) | WB (1:1000) |
| Rabbit anti-MFN1 | Cell Signaling (# 14739S) | IHC (1:200), IF (1:200)  WB (1:1000) |
| Rabbit anti-MFN2 | Abcam (ab56889) | WB (1:1000) |
| Mouse anti-β-Actin | Abcam (ab8226) | WB (1:1000) |
| Rabbit anti-P16 ^INK4a^ | Cell Signaling (#29271) | IHC (1:200), IF (1:100) |
| Rabbit anti-P21 | Santa Cruz (sc-397) | IHC (1:200), IF (1:50) |
| Rabbit anti-ADAMTS5 | Abcam (ab41037) | IHC (1:400), WB (1:1000) |
| Rabbit anti-PGC-1α | Abcam (ab191838) | WB (1:1000) |
| Rabbit anti-TFAM | Abmart (TA0531) | WB (1:1000) |
| Rabbit anti-NRF1 | Abmart (T57168) | WB (1:1000) |
| Rabbit anti-PCNA | Proteintech (10205-2-AP) | IHC (1:400), IF (1:200) |
| Rabbit anti-Bcl2 | Cell Signaling (#3498) | WB (1:1000) |
| Rabbit anti-TOMM20 | Servicebio (GB111481) | IF (1:200) |
| Rabbit anti-ERK1/2 | Cell Signaling (#4695) | WB (1:1000) |
| Rabbit anti-p-ERK1/2  (Thr202/Tyr204) | Cell Signaling (#4370) | WB (1:1000) |
| Rabbit anti-JNK | Cell Signaling (#9252) | WB (1:1000) |
| Rabbit anti-p-JNK  (Thr183/Tyr185) | Cell Signaling (#4668) | WB (1:1000) |
| Rabbit anti-p38 | Cell Signaling (#8690) | WB (1:1000) |
| Rabbit anti-p-p38  (Thr180/Tyr182) | Cell Signaling (#4511) | WB (1:1000) |
| Rabbit anti-IgG  isotype control | Cell Signaling (#49077) | / |
| Mouse anti-IgG  isotype control | Abcam (ab37355) | / |
| Alexa Fluor 488  Goat Anti-Rabbit IgG | Jackson ImmunoResearch  (111-545-003) | IF (1:500) |
| Alexa Fluor 594  Goat Anti-Mouse IgG | Jackson ImmunoResearch  (115-585-003) | IF (1:500) |
| Alexa Fluor 488  Donkey Anti-Mouse IgG | Invitrogen (A-21202) | IF (1:1000) |
| Alexa Fluor 594  Donkey Anti-Rabbit IgG | Invitrogen (A-21207) | IF (1:1000) |
| Peroxidase AffiniPure Goat Anti-Rabbit lgG | Yeasen (33101ES60) | WB (1:10000)  IHC (1:500) |
| Peroxidase AffiniPure Goat Anti-Mouse IgG | Yeasen (33201ES60) | WB (1:10000)  IHC (1:500) |
