# Supplementary figures and images for: Dimeric PKM2 in chondrocytes impairs mitochondrial homeostasis in osteoarthritis
Source: Cell Death Dis. 2026 Mar 25;17(1):370. doi: 10.1038/s41419-026-08621-4 (PMC13039331; doi:10.1038/s41419-026-08621-4)

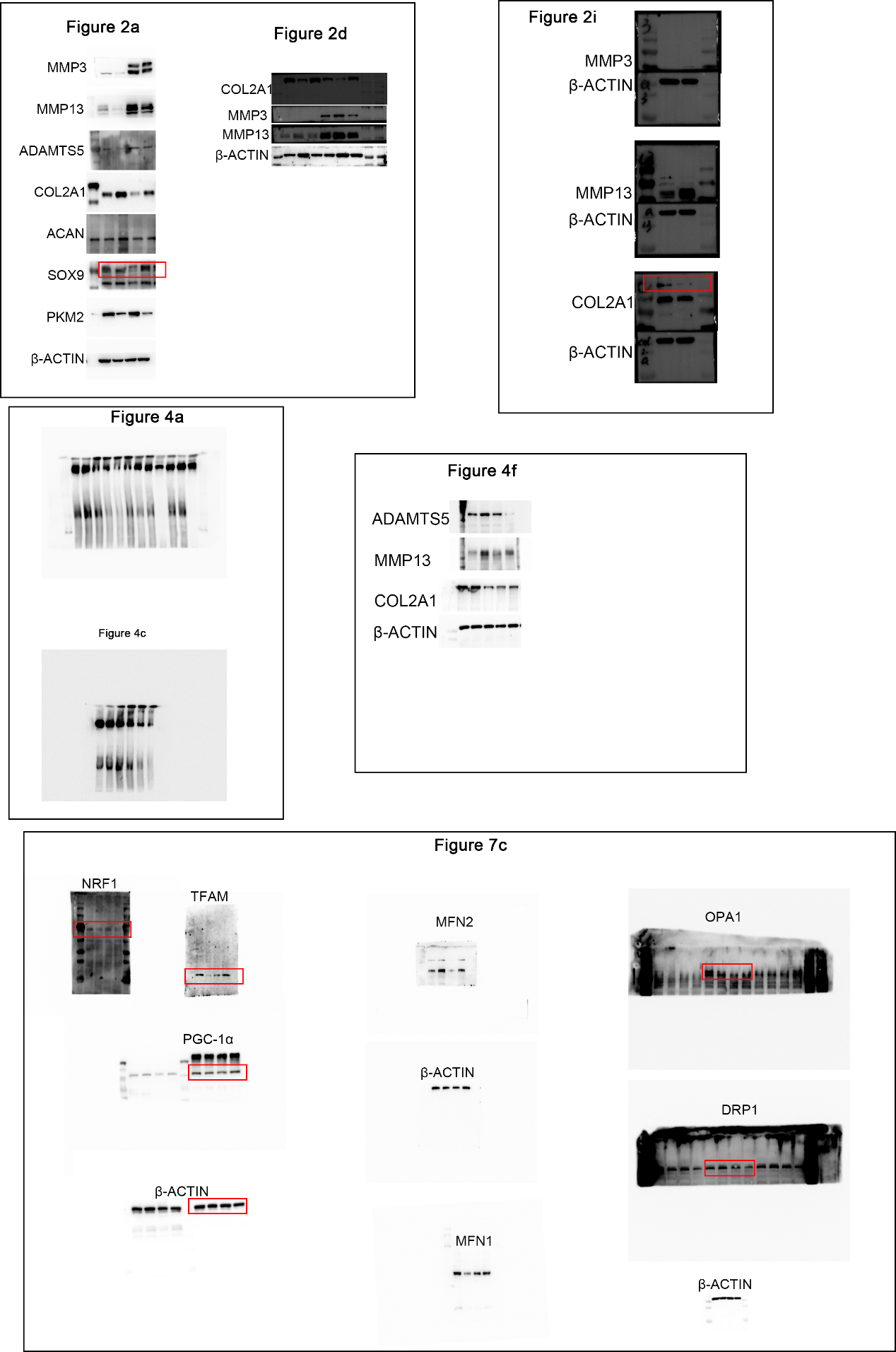

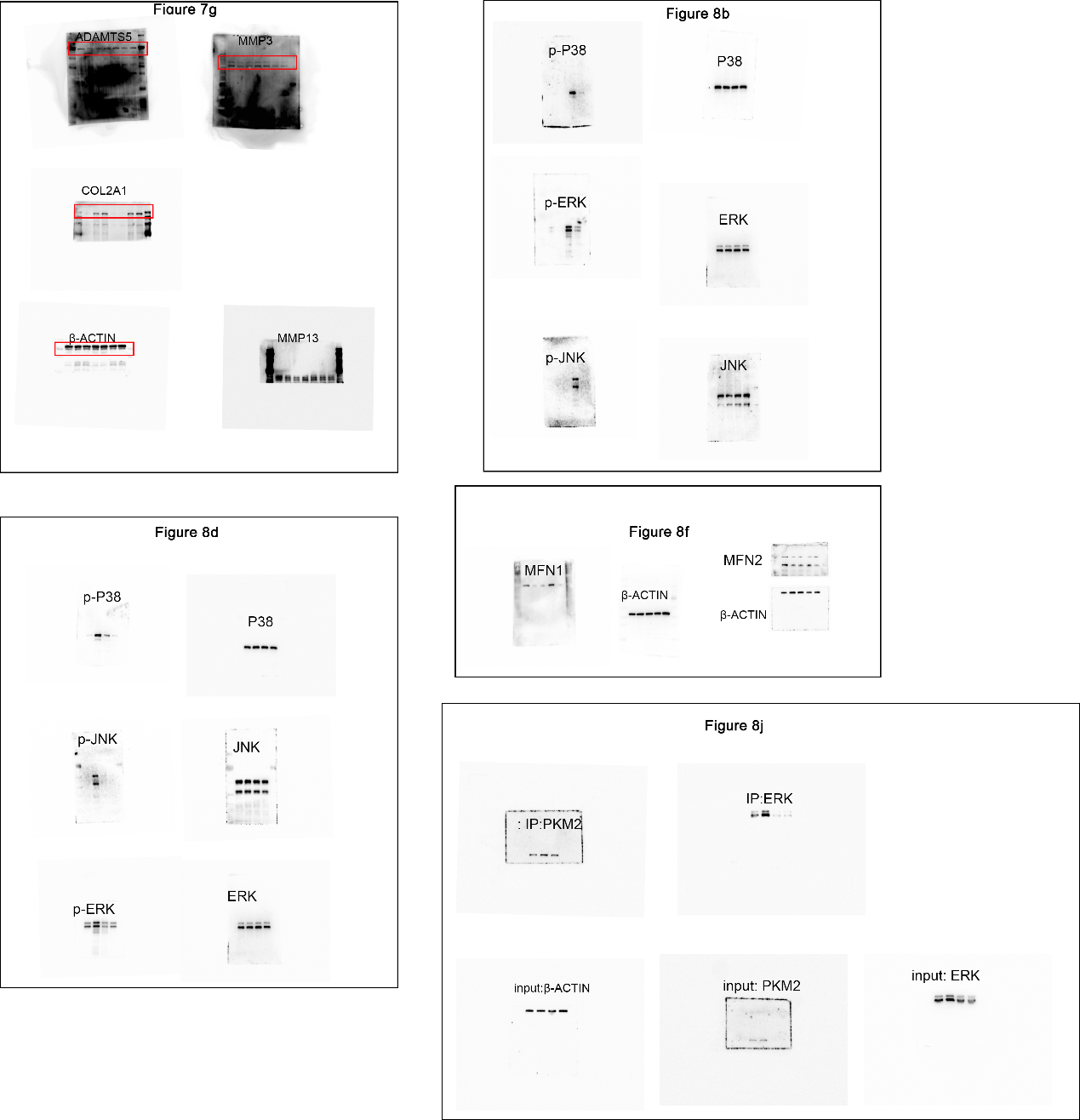

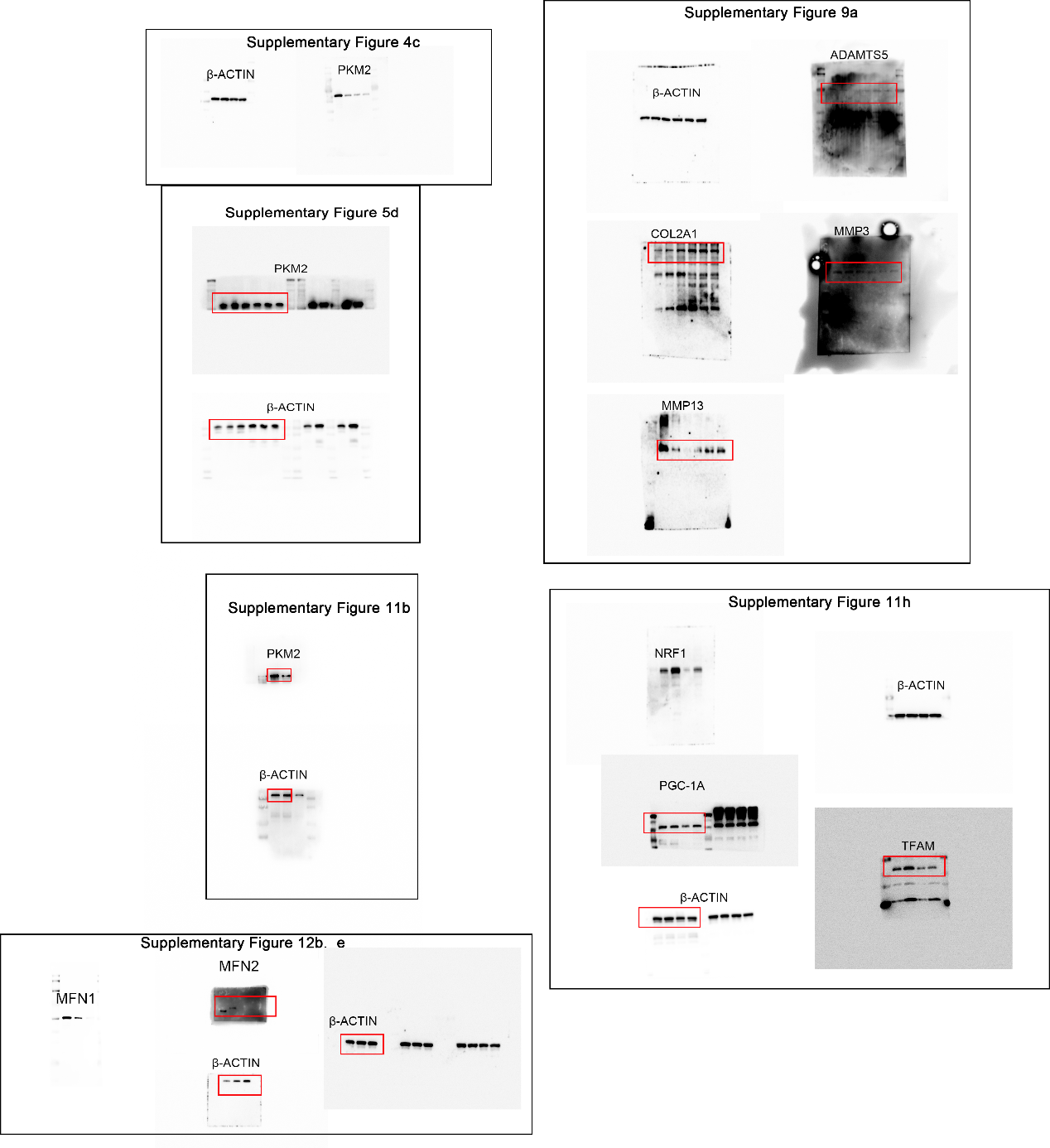

Supplement: Supplementary file 2 — Original Western blots [file 41419_2026_8621_MOESM2_ESM.docx]
